# Time-resolved and theoretical analysis of Mo-carbene transformations in metathesis of ethylene with 2-butene†

**DOI:** 10.1039/d4sc06833e

**Published:** 2025-01-08

**Authors:** Tatiana Otroshchenko, Aleksandr Fedorov, Qiyang Zhang, David Linke, Jarosław Handzlik, Mirjam Schröder, Björn Corzilius, Evgenii V. Kondratenko

**Affiliations:** a Leibniz-Institut für Katalyse e.V. Albert-Einstein-Str. 29a D-18059 Rostock Germany tatiana.otroshchenko@catalysis.de evgenii.kondratenko@catalysis.de; b Cracow University of Technology, Faculty of Chemical Engineering and Technology ul. Warszawska 24 31-155 Kraków Poland; c Institute of Chemistry, University of Rostock Albert-Einstein-Str. 27 18059 Rostock Germany; d Department Life, Light & Matter, University of Rostock Albert-Einstein-Str. 25 18059 Rostock Germany

## Abstract

Although supported Mo-containing catalysts have been extensively investigated in the metathesis of ethylene with 2-butene to propene, the mechanisms of the formation and transformation of catalytically active Mo-carbenes in the course of the reaction are still not fully understood. The difficulties arise because only a tiny fraction of MoO_*x*_ species can form Mo-carbenes *in situ*, making the detection of the latter by spectroscopic means very unlikely. Herein, purposefully designed steady-state and transient experiments including their kinetic evaluation and density functional theory calculations enabled us to elucidate mechanistic and kinetic details of the above reaction-induced processes in the metathesis reaction over a Mo/P/SiO_2_ catalyst at 50 °C. We established that, in parallel with the desired reaction cycle, molybdacyclobutanes also undergo reversible structural transformations which might be one of the reasons for low steady-state catalyst activity. Based on the results obtained, strategies for controlling the concentration of the inactive species and accordingly catalyst activity have been suggested and experimentally validated.

## Abstract 2

Novel insights into formation and transformation of Mo-carbenes in metathesis of ethylene with 2-butene are provided. Mo-carbenes are involved in the metathesis cycle and can be converted into “inactive” complex formed with participation of butene.

## Graphical abstract

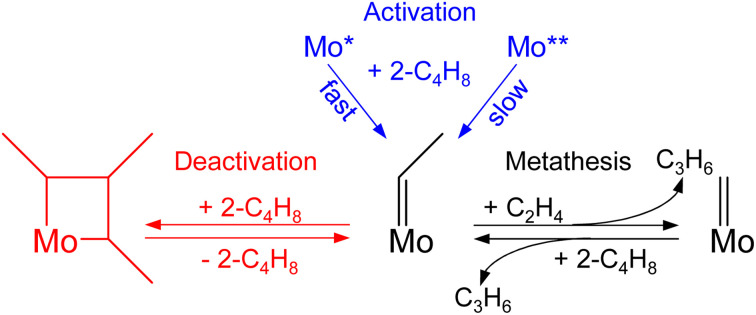

## Introduction

Metathesis of ethylene with 2-butene is a large-scale process used for the on-purpose production of propene, the second most widely produced olefin in the chemical industry.^1,2^ Catalysts based on oxides of W, Mo or Re dispersed on high-surface-area supports are used for this reaction.^3,4^ According to the classical mechanism elucidated by Hérisson and Chauvin,^5,6^ surface metal-carbenes (M=CH-R, where M is W, Mo or Re), which serve as active sites, react with ethylene or 2-butene to form metallacyclobutane intermediates decomposing to gas-phase propene. The M=CH-R species are formed *in situ* as a result of the interaction of MO_*x*_ with olefins. Therefore, the knowledge of the mechanisms of M=CH-R formation and, especially, undesired transformations under the reaction conditions is very important to control the concentration of the active species and accordingly the propene production through catalyst design or modulating the reaction conditions.

It has been shown that the presence of hydroxyl species on the catalyst surface,^7–13^ the usage of metathesis inactive cocatalysts,^14–16^ or a certain catalyst treatment (photoreduction in CO followed by treatment in cyclopropane;^17^ reduction with organosilicon reductants;^18^ high-temperature treatment in CH_4_;^19^ propene adsorption – desorption treatment;^9^ treatment in methanol;^8^*etc.*) facilitate the transformation of MO_*x*_ into M=CH-R. Although the treated catalysts demonstrate high initial metathesis activity, they rapidly deactivate with time on stream and achieve a steady-state activity like their untreated counterparts. The reason for such deactivation seems to be a decrease in the concentration of M=CH-R due to their low stability^8,17,20^ and/or transformation of the metallacyclobutane intermediates into stable inactive complexes as theoretically suggested for ethylene metathesis.^21^ Because the fraction of MO_*x*_ species capable of forming M=CH-R is extremely low,^8,16,22,23^ state-of-the-art spectroscopic methods typically do not provide direct insight into the formation and further conversion of carbenes.

The above challenges and fundamental gaps motivated us to perform sophisticated steady-state and transient catalytic tests including their kinetic evaluation as well as density functional theory (DFT) calculations. The Mo/P/SiO_2_ catalyst, which was introduced in our previous study^10^ and shows exceptional activity even at 50 °C, was used in the present study. Special emphasis was placed on the analysis of both selective pathways in the classical Chauvin cycle and side reactions of M=CH-R. The results obtained provide the basis for improving the catalyst activity through modulating the reaction conditions.

## Results and discussion

### Structural catalyst properties

The Mo/P/SiO_2_ catalyst described in ref. 10 was used for kinetic tests at 50 °C in the metathesis of ethylene with 2-butenes using different reaction feeds under conditions free of heat and mass transport limitations. Its unpromoted counterpart, Mo/SiO_2_, shows too low activity at 50 °C and was not used in the kinetic tests. Nevertheless, important structural properties of these catalysts are presented and discussed for comparative purposes. The UV-vis spectra of both samples are characterized by strong absorption bands at around 250 and 290 nm and shoulders at around 330 and 400 nm (Fig. S1(a)†). These bands can be assigned to the ligand-to-metal charge transfer transitions in tetrahedral and octahedral MoO_*x*_ species, as well as in crystalline MoO_3_.^13^ A straightforward assignment of the band position to the specific Mo-containing species is however complicated due to significant overlapping of the absorption bands. The promoter P seems to lead to a slight decrease in the degree of oligomerization of MoO_*x*_ species as concluded from the UV-vis edge energy (*E*_g_) determined from the Tauc plot (Fig. S1(b)†).

The effect of P on the oligomerization degree of supported MoO_*x*_ may be explained as follows. The promotion of SiO_2_ with P leads to the creation of a new type of OH site characterized by a sharp band at 3667 cm^−1^ (Fig. S1(c),† red dashed line). These sites should be involved in anchoring MoO_*x*_ because the ratio of the intensity of this band to that at 3747 cm^−1^ (related to isolated silanols) for Mo/P/SiO_2_ is lower in comparison with that for P/SiO_2_. After deposition of MoO_*x*_ species on the support, a new broad band centred at about 3627 cm^−1^ appears (Fig. S1(c),† solid lines), which can be assigned to O–H stretching vibrations in molybdenol groups (Mo–OH) involved in hydrogen bonding to neighbouring oxygen or to silanol groups interacting with oxygen atoms of MoO_*x*_ species.^9^

The Raman spectra of both Mo/SiO_2_ and Mo/P/SiO_2_ are characterized by the bands at 814 and 990 cm^−1^ originating from MoO_*x*_ and/or MoO_3_ species (Fig. S1(d)†).^24^ The Raman spectrum of Mo/P/SiO_2_ additionally contains a weak band at 877 cm^−1^, which can be assigned to the P–O stretching vibrations in phosphates (*ν*_s_P(OH), *ν*_s_P(OH)_2_, and/or *ν*_s_P(OH)_3_).^25^

The chemical nature of P-containing species was further analysed by XPS (Fig. S1(e)†) and ^31^P NMR (Fig. S1(f)†). A broad XPS peak in the P 2p region, which can be deconvoluted into a doublet (134.9 eV, 135.8 eV), can be assigned to the surface PO_4_^3−^ species.^26–28^ No features of metal phosphides (binding energy of ∼129.5 eV) can be observed. The ^31^P MAS-NMR spectrum of the reference P/SiO_2_ shows four signals at 0.4, −6.1, −10.4 and −23.1 ppm. The first three signals likely belong to single isolated phosphate groups (Si–O–P=O–(OH)_2_), while the last signal indicates the presence of phosphate species with a bridged structure ((Si–O)_2_–P=O–OH).^29^ For the Mo/P/SiO_2_ sample, the signal at ∼0 ppm is upfield shifted by 3 ppm and broadened, hinting at contact or pseudo-contact interactions with the metal. The signals at −10.4 and −23.1 ppm remain unchanged in their chemical shift.

In summary, highly dispersed and polymerized tetrahedral and octahedral MoO_*x*_ as well as crystalline MoO_3_ are present on the surface of the catalysts. The latter species unlikely participate in the formation of active metal-carbenes^23^ and therefore should not play any role in the metathesis reaction. From the above-shown data we can conclude that the majority of P exists as monomeric phosphate species which likely participate in the anchoring of non-crystalline MoO_*x*_ sites.

### Catalytic activity in C_2_H_4_ – *trans*-2-C_4_H_8_ metathesis

**Fig. 1:**
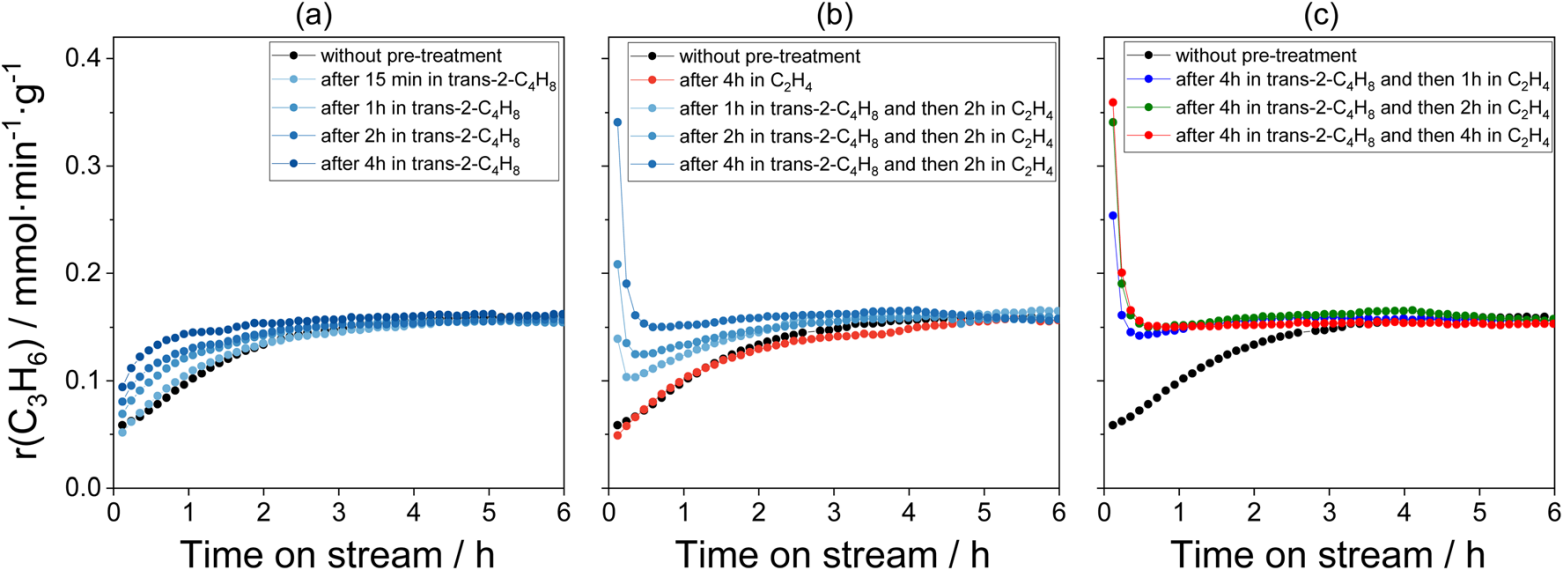
Temporal changes of the rate of propene formation determined over calcined Mo/P/SiO_2_ (marked in black in all figures) and Mo/P/SiO_2_ after various treatment procedures: (a) after treatment in *trans*-2-C_4_H_8_ for different durations of time; (b) after treatment in C_2_H_4_ for 4 h and after treatment in *trans*-2-C_4_H_8_ for different durations of time followed by treatment in C_2_H_4_ for 2 h; (c) after treatment in *trans*-2-C_4_H_8_ for 4 h followed by treatment in C_2_H_4_ for different durations of time. Treatments and metathesis reaction were carried out at 50 °C.

After treatment of the Mo/P/SiO_2_ catalyst in air at 500 °C for 3 h and cooling down to 50 °C in nitrogen, an industrially relevant C_2_H_4_/*trans*-2-C_4_H_8_/N_2_ = 5/5/1 feed was added. The rate of propene formation increased more than two times from an initial (determined after 7 min on reaction stream) non-zero value with increasing time on stream and reached a steady-state value after about 4 h on stream (black symbols in Fig. 1). The increase may be due to the reaction-induced conversion of MoO_*x*_ to catalytically active Mo-carbenes. According to some previous studies,^10,11^ different olefins have different reactivity in this respect. With this in mind, we treated the Mo/P/SiO_2_ catalyst at 50 °C in *trans*-2-C_4_H_8_, C_2_H_4_, or *trans*-2-C_4_H_8_ followed by C_2_H_4_ for different durations of time before starting the metathesis reaction at the same temperature. The same Mo/P/SiO_2_ catalyst sample (initially tested in the metathesis of ethylene with 2-butenes at 50 °C for 10 h and regenerated in air at 500 °C for 3 h) was applied for all experiments.

The catalyst treatment in *trans*-2-C_4_H_8_ increased the initial rate of propene formation but did not influence the steady-state rate (Fig. 1(a)). Moreover, the time required to reach steady-state operation decreased with increasing treatment time. These changes may be related to an increase in the concentration of Mo-carbenes during the catalyst treatment.

It is worth mentioning that propene and pentenes were observed during the treatment of Mo/P/SiO_2_ in *trans*-2-C_4_H_8_ (Fig. S2(a)†). Their appearance is more likely due to the metathesis of *trans*-2-C_4_H_8_ with 1-C_4_H_8_ formed through isomerization of *trans*-2-C_4_H_8_ which is typical for Mo/SiO_2_ catalysts.^30^ According to this reaction, propene and pentene are formed in a molar ratio of 1 : 1. A similar value was determined for the ratio of the formation rates of these olefins during the catalyst treatment in *trans*-2-C_4_H_8_ (Fig. S2(a)†).

The catalyst treatment in C_2_H_4_ at 50 °C for 4 h did not significantly affect the initial rate of propene formation and its time-on-stream behaviour when compared to the results obtained with the untreated Mo/P/SiO_2_ (Fig. 1(b)). According to previous DRIFTS and C_2_H_4_-TPD data,^10^ C_2_H_4_ hardly interacts with the oxidized catalyst at 50 °C and does not yield any surface hydrocarbon species. Thus, in contrast to the treatment in *trans*-2-C_4_H_8_, the treatment in C_2_H_4_ does not result in the formation of Mo-carbenes from MoO_*x*_.

The initial rate of propene formation over the catalyst treated first in *trans*-2-C_4_H_8_ and then in C_2_H_4_ (Fig. 1(b) and (c)) is significantly higher than that of its untreated counterpart or treated either in *trans*-2-C_4_H_8_ or in C_2_H_4_. The highest activity was determined after 4 h treatment in *trans*-2-C_4_H_8_ followed by 4 h treatment in C_2_H_4_. It was more than six times higher than the rate determined over the calcined untreated catalyst and more than two times higher than the steady-state value. This high activity, however, decreased rapidly within the first hour on reaction stream regardless of the treatment duration. The time required to reach the steady-state activity increased as the treatment time in *trans*-2-C_4_H_8_ decreased. Noticeably, such behaviour is unlikely related to the presence of phosphorus in the catalyst since similar temporal changes of the rate of propene formation were observed for the unpromoted Mo/SiO_2_ catalyst (Fig. S3†).

No gaseous products (except traces of C_3_H_6_ due to the presence of tiny amounts of *trans*-2-C_4_H_8_ in the line) were observed during the treatment of Mo/P/SiO_2_ in C_2_H_4_ after the treatment in *trans*-2-C_4_H_8_ (Fig. S2(b)†). Such result implies that no products are formed during the interaction of C_2_H_4_ with the catalyst pre-activated in *trans*-2-C_4_H_8_ or their concentration is below the detection limit of the on-line GC used.

To check if the propene formation rate can be influenced by short-term treatment of the catalyst in ethylene, we performed a control experiment. The Mo/P/SiO_2_ sample was first treated in *trans*-2-C_4_H_8_ for 1 h and then in C_2_H_4_ for 30 min. Hereafter, the catalyst was exposed to the standard reaction feed (C_2_H_4_/*trans*-2-C_4_H_8_/N_2_ = 5/5/1). After some time on this stream, the catalyst was treated in C_2_H_4_ for short intervals (3 min) followed by feeding the reaction mixture. The catalyst activity increased by about 30% after each treatment in C_2_H_4_ (Fig. S4†) and then decreased rapidly with increasing time on the metathesis stream.

### Determination of the steady-state concentration of Mo-carbenes

**Fig. 2:**
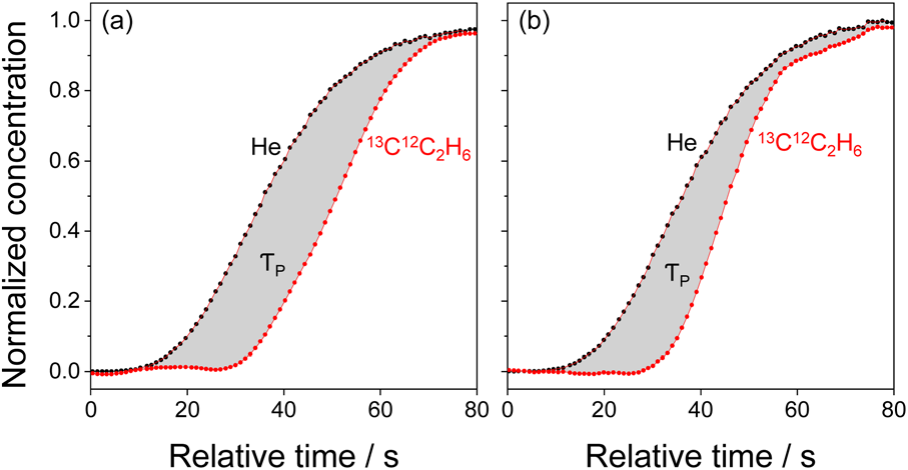
Normalized responses of ^13^C^12^C_2_H_6_ and He (inert tracer) as a function of relative time (time after the switch of non-labeled feed to ^13^C-labeled feed) obtained for (a) calcined untreated Mo/P/SiO_2_ and (b) Mo/P/SiO_2_ sequentially treated in *trans*-2-C_4_H_8_ at 50 °C for 4 h and in C_2_H_4_ at 50 °C for 4 h.

Motivated by our previous study,^16^ we applied the Steady-State Isotopic Transient Kinetic Analysis (SSITKA) method^31,32^ to verify whether the increase in the rate of propene formation after a two-step treatment of the catalyst in *trans*-2-C_4_H_8_ and then in C_2_H_4_ was due to the increase in the concentration of Mo-carbenes. In these tests carried out with untreated Mo/P/SiO_2_ and its counterpart initially treated in *trans*-2-C_4_H_8_ for 4 h and then in C_2_H_4_ for 4 h, we started with a non-labelled reaction feed (C_2_H_4_/*trans*-2-C_4_H_8_/Ar) to reach a steady-state operation (after about 20 min on stream). Hereafter, this feed was replaced by a feed containing ^13^C_2_H_4_ (^13^C_2_H_4_/*trans*-2-C_4_H_8_/He/Ar; He was used as an inert tracer). Importantly, the concentrations of ethylene and 2-butene in these feeds were the same. The replacement of ^12^C_2_H_4_ by ^13^C_2_H_4_ in the reaction feed did not lead to a change in the rate of propene formation (confirmed by on-line GC measurements). Thus, a kinetic isotope effect does not play a significant role because of the small difference in the atomic masses of ^12^C and ^13^C. The concentration of ^13^C^12^C_2_H_6_ increased with time on stream after switching to the labelled feed. The normalized (with respect to the steady-state signals) responses of ^13^C^12^C_2_H_6_ and He (inert tracer) obtained in the SSITKA tests are shown in Fig. 2. Based on the theory of the SSITKA method,^31,32^ the surface residence time (*τ*_P_) of Mo-carbenes leading to propene was calculated from the area between the transient responses of He and ^13^C^12^C_2_H_6_ (Fig. 2, Table S1†). Their concentration (*N*_P_) was obtained from eqn (1). It is noteworthy that the fraction (*N*_P_ divided by the total amount of Mo) of MoO_*x*_ resulting in Mo-carbenes does not exceed 0.1% (Table S1†).

1 *N*_P_ = *τ*_P_ × *r*(C_3_H_6_),

where *r*(C_3_H_6_) is the steady-state rate of propene formation determined at the time of switching.

The *N*_P_ value determined for the untreated Mo/P/SiO_2_ is more than seven times lower than that for the Mo/P/SiO_2_ treated first in *trans*-2-C_4_H_8_ and then in C_2_H_4_. We used these values and the rate of propene formation over these catalysts to calculate the turnover frequency of propene formation (TOF) according to eqn (2).

2 TOF = *r*(C_3_H_6_)/*N*_P_

No obvious difference was found (Table S1†). Thus, the catalyst treatment in *trans*-2-C_4_H_8_/C_2_H_4_ seems to increase the concentration of Mo-carbenes but not to create other highly reactive Mo-carbenes.

### Catalyst activity in the presence of 1-C_4_H_8_

We also performed the metathesis reaction in the presence of 5 vol% of 1-C_4_H_8_ to understand if and how this olefin affects the catalyst activity. Propene, pentenes, and trace amounts of hexenes were detected as reaction products through various metathesis reactions shown in eqn (3)–(5).

3 C_2_H_4_ + 2-C_4_H_8_ ⇄ 2C_3_H_6_

4 2-C_4_H_8_ + 1-C_4_H_8_ ⇄ C_3_H_6_ + C_5_H_10_

5 1-C_4_H_8_ + 1-C_4_H_8_ ⇄ C_2_H_4_ + C_6_H_12_

**Fig. 3:**
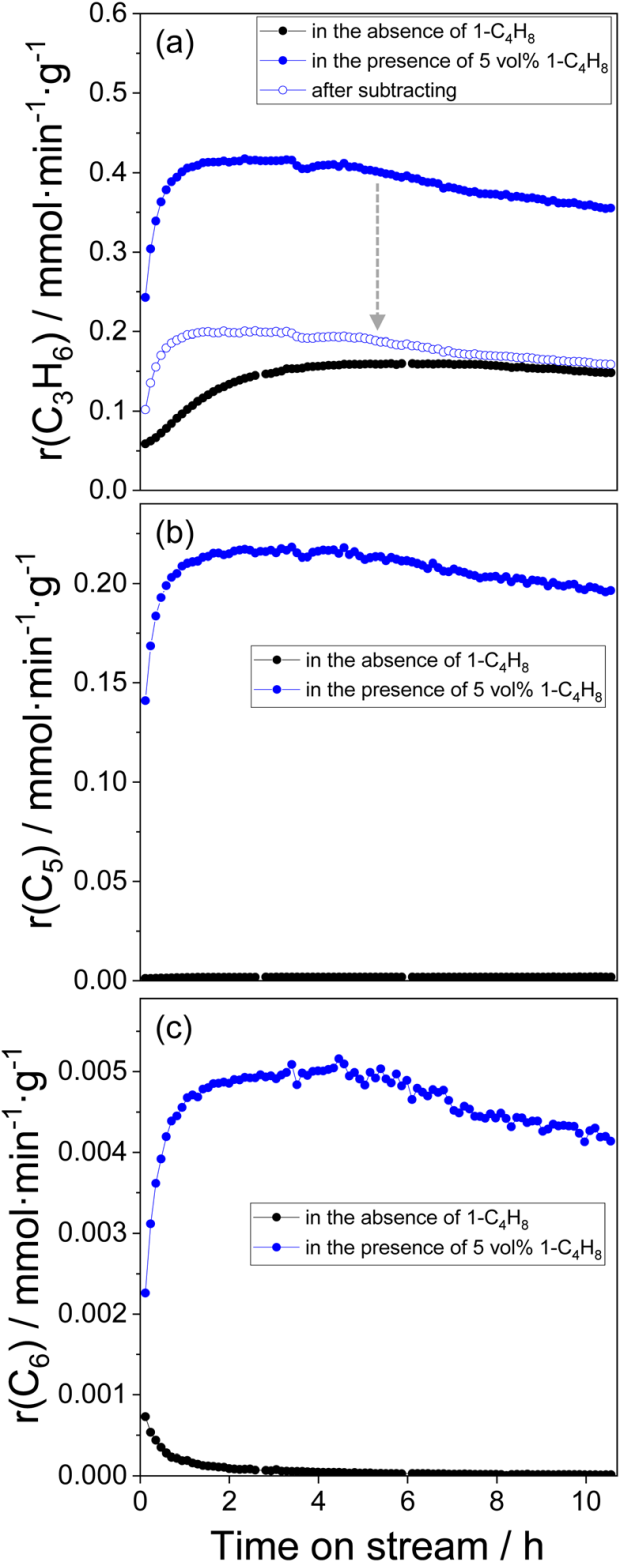
Temporal changes of the rates of formation of (a) propene, (b) pentenes, and (c) hexenes determined at 50 °C over calcined Mo/P/SiO_2_ by using reaction mixture C_2_H_4_/*trans*-2-C_4_H_8_/N_2_ = 10/10/2 (the data are marked in black in all figures) and by using reaction mixture C_2_H_4_/*trans*-2-C_4_H_8_/1-C_4_H_8_/N_2_ = 10/9/1/2 (the data are marked in solid blue in all figures). Open blue circles in (a) are related to the data obtained after subtracting the contribution of *trans*-2-C_4_H_8_ – 1-C_4_H_8_ metathesis in propene formation.

The temporal changes of the rates of formation of propene, pentenes, and hexenes are shown in Fig. 3(a), (b), and (c) respectively, together with the corresponding data obtained in the test performed without co-fed 1-C_4_H_8_. All rates in the presence of 1-C_4_H_8_ gradually increased with time on stream during the first hour on reaction stream and started to decrease after about 4 h on stream. The initial rate of propene formation was about four times and the final rate (after about 10.5 h on stream) was more than two times higher than the corresponding rates determined in the absence of 1-C_4_H_8_. According to eqn (3)–(5), propene is formed *via* metathesis reactions of C_2_H_4_ with *trans*-2-C_4_H_8_ and *trans*-2-C_4_H_8_ with 1-C_4_H_8_. Knowing the rate of pentene formation (Fig. 3(b)), we can estimate the contribution of the butene metathesis (eqn (4)) to the formation of propene. Since the reaction gives propene and pentene in the molar ratio of 1 : 1, the rate of propene formation should be equal to the rate of pentene formation. By subtracting this value from the total rate of propene formation, we can roughly estimate the rate of propene formation through the metathesis of C_2_H_4_ with *trans*-2-C_4_H_8_ (Fig. 3(a), open blue circles). In comparison with the control test without co-fed 1-C_4_H_8_, the obtained initial rate is higher, and the induction period characterized by an increase in this rate with time on stream is shorter. This result implies that 1-C_4_H_8_ accelerates the formation of Mo-carbenes active in the metathesis of C_2_H_4_ with *trans*-2-C_4_H_8_.

### Formation and transformation of Mo-carbenes

According to the classical Chauvin mechanism of the metathesis of ethylene with 2-butenes, the main reaction steps are described by eqn (6) and (7). The rate of propene formation is influenced by the concentration of Mo-carbenes and their intrinsic activity. These species must be formed *in situ* through the reaction of supported MoO_*x*_ with fed olefins.^11,23,33–35^6
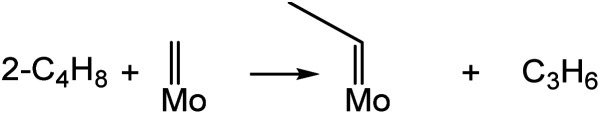
7
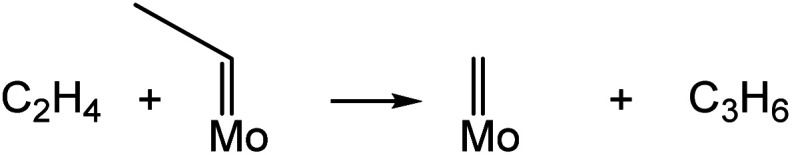


Accordingly, the gradual increase in the rate of propene formation over untreated Mo/P/SiO_2_ with time on reaction stream (Fig. 1, black symbols) can be related to the increase in the number of active Mo-carbenes. When the rate is extrapolated to zero time on stream, a non-zero value is obtained. Therefore, it is reasonable to assume that there are at least two types of MoO_*x*_ species differing in their reactivity to yield Mo-carbenes. These species may differ in their molecular structure, location, and/or oxidation state of Mo. The initial activity of Mo/P/SiO_2_ should be related to the presence of MoO_*x*_ species (Mo*) that quickly react with *trans*-2-C_4_H_8_ to form Mo-carbenes. Notably, C_2_H_4_ is unable to generate the latter species at 50 °C.^10^ Another type of MoO_*x*_ species (Mo**) is characterized by a lower reactivity to form Mo-carbenes as reflected by the slow increase in the rate of propene formation with increasing time on stream (Fig. 1, black symbols).

**Fig. 4:**
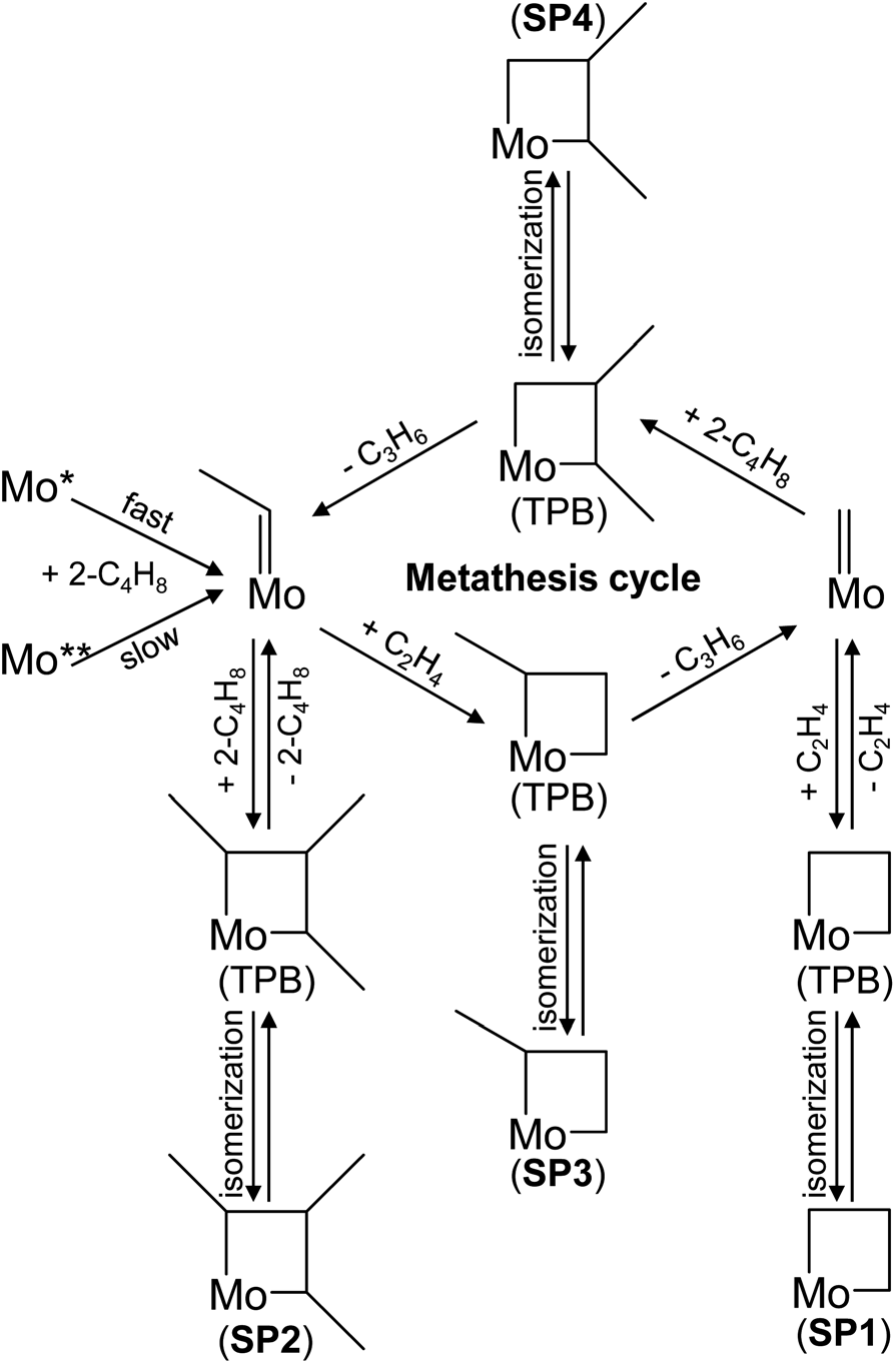
Schematic illustration of Mo-carbene formation from Mo* and Mo** species, metathesis cycle, and the formation of “inactive” complexes (square pyramidal (SP) molybdacyclobutanes).

Apart from their formation, the desired Mo-carbenes may be involved in side reactions responsible for the decrease in the rate of propene formation over the catalyst treated initially in *trans*-2-C_4_H_8_ followed by C_2_H_4_ (Fig. 1(b) and (c)). As the latter olefin activates the catalyst treated in *trans*-2-C_4_H_8_, we suggest that *trans*-2-C_4_H_8_ may “deactivate” the active Mo-carbenes through the formation of an “inactive” complex that does not participate in the formation of propene. This complex can be a cyclic compound due to the following arguments. The cycloaddition of an olefin molecule to a carbene site, resulting in the formation of a molybdacyclobutane complex, is the first step in the metathesis catalytic cycle.^6,36^ Theoretical studies of ethylene metathesis over MoO_*x*_/Al_2_O_3_ (ref. 21, 37 and 38) and MoO_*x*_/SiO_2_ (ref. 36 and 39) have shown that ethylene can react with Mo-methylidene (Mo=CH_2_) to give a molybdacyclobutane complex with a trigonal bipyramidal (TBP) geometry. This intermediate can react back to Mo=CH_2_ and C_2_H_4_ or be converted into its inactive isomer with a square pyramidal (SP) geometry (structure SP1 in Fig. 4). This rearrangement was considered to be one of the reasons for catalyst deactivation during ethylene metathesis.^33,39^ By analogy, we put forward that such undesired transformations of molybdacyclobutane complexes can also occur in the course of the ethylene – 2-butene metathesis (structures SP2–SP4 in Fig. 4). To check this hypothesis, DFT calculations were performed.

**Fig. 5:**
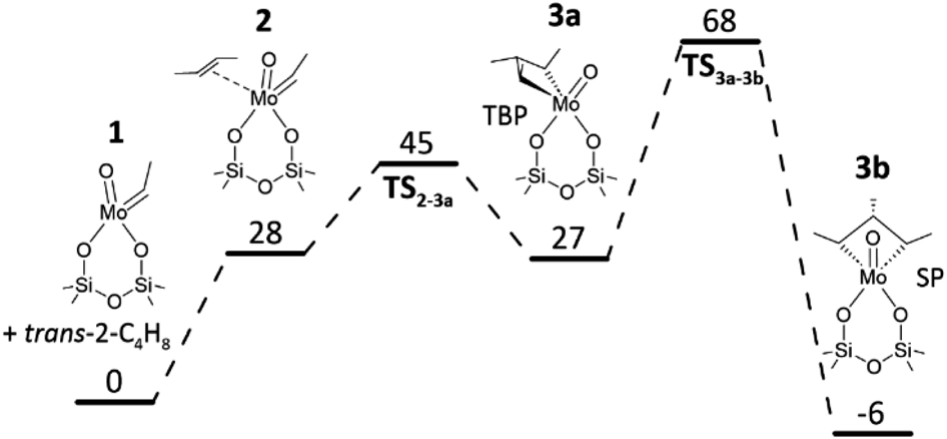
Gibbs energy profile (kJ mol^−1^) at 50 °C for a reaction between *syn*-Mo(vi) ethylidene species and *trans*-2-C_4_H_8_.

The calculated pathways of the reaction between Mo=C_2_H_4_ and *trans*-2-C_4_H_8_ are presented in Fig. 5. The formation of the trisubstituted molybdacyclobutane complex 3a with a TBP geometry is endergonic, but the predicted overall activation Gibbs energy for this step is quite low (45 kJ mol^−1^). The thermodynamically unstable intermediate 3a should rapidly decompose to the reactants in a reverse step with a very low Gibbs energy barrier of 18 kJ mol^−1^. The isomerization of the TBP molybdacyclobutane 3a to the thermodynamically stable SP molybdacyclobutane 3b is another possibility, requiring a higher energy barrier to overcome, but it still may proceed effectively at 50 °C. The reverse transformation of the SP isomer to the TBP one will occur slower due to a relatively high activation Gibbs energy for this step (74 kJ mol^−1^). Therefore, the SP molybdacyclobutane 3b can be considered as a resting state of the metathesis process, *i.e.*, the postulated “inactive” complex. The equilibrium constant for its formation from Mo=C_2_H_4_ and *trans*-2-C_4_H_8_ was estimated to be around 8.1.

Stable SP molybdacyclobutane complexes can be also formed along the productive metathesis reactions (7) and (6) (Fig. S5 and S6,† respectively). However, the calculated activation barriers for the rearrangement of the monosubstituted and disubstituted SP molybdacyclobutanes to their TBP isomers (60 and 68 kJ mol^−1^, respectively) are lower than in the case of 3b (74 kJ mol^−1^) bearing three methyl substituents in the ring. Hence, for the latter the equilibrium shift towards the formation of Mo-carbenes after changing the olefin feed to ethylene will be slower. This is especially seen if compared to the monosubstituted SP molybdacyclobutane formed from ethylene and Mo=C_2_H_4_ (Fig. S5†). This can explain the observed increase of the metathesis activity after final ethylene treatment, in contrast to the standard pretreatment with 2-butene (Fig. 1). Nevertheless, based on the predicted activation Gibbs energies, the SP–TBP interconversion should be much faster than the formation of the active sites from MoO_*x*_ species.^11^

### Kinetic analysis of the ethylene – 2-butene metathesis

**Fig. 6:**
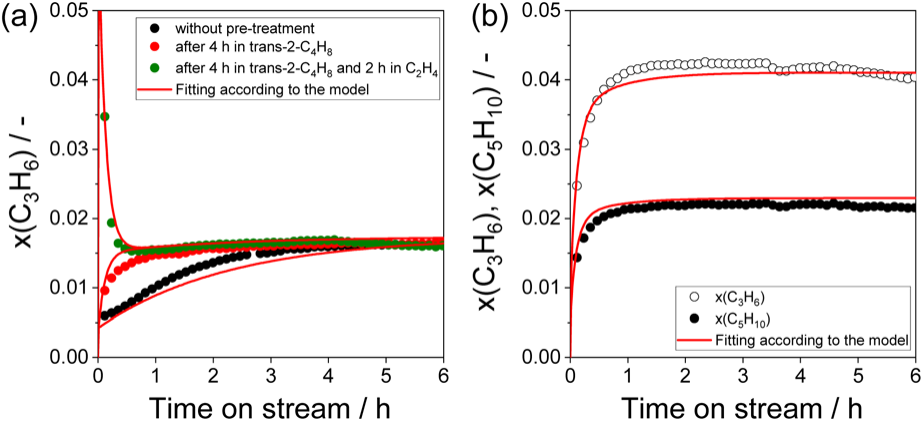
Experimental and fitting data representing time-on-stream changes of (a) the propene molar fraction during metathesis of ethylene with 2-butene over calcined Mo/P/SiO_2_ and over that after different treatments, (b) propene and pentene molar fractions during metathesis of ethylene with 2-butene in the presence of 5 vol% 1-C_4_H_8_ over calcined Mo/P/SiO_2_.

Based on the above-discussed experimental observations we developed a kinetic model considering reaction pathways responsible for the *in situ* formation of Mo-carbenes as well as for their desired reactions in the course of the metathesis of ethylene and 2-butene and side transformations into inactive intermediates (Table S2†). The obtained kinetic parameters (reaction rate and equilibrium constants) are given in Table S3.† The model was fit to all experimental data from Fig. 1 and 3 simultaneously. It describes the temporal profiles of propene formed over differently treated Mo/P/SiO_2_ (Fig. 6(a) and S7(a) and (c)†). The profiles of propene and pentenes formed during the experiment performed in the presence of 5 vol% 1-C_4_H_8_ were also described by this model (Fig. 6(b)).

The overall comparison of the values of the key kinetic parameters estimated by DFT and data-driven kinetic modelling is presented in Table S4.† The value of equilibrium constant K_3_ (see Table S3†) describing the formation of an “inactive” complex (*trans*-2-C_4_H_8_ + Mo=C_2_H_4_ ⇄ Mo=C_6_H_6_) was around 35.6 according to the developed kinetic model. This value can be compared with the value of 8.1 obtained from DFT calculations. The agreement is reasonable, taking into account the limited accuracy of the DFT methods and the exponential dependence of the equilibrium constant on the reaction Gibbs energy. It is worth mentioning that the ratio of the rate constants of reactions 2.1 and 2.2 (Table S2, Fig. S5 and S6†), evaluated from the overall Gibbs energy barriers (TBP–SP isomerization step is omitted), is 13, which is in qualitative accordance with the kinetic modelling results (*k*_2.1_/*k*_2.2_ = 4.5, Table S3†). The conversion of Mo=C_2_H_4_1 and ethylene to Mo=CH_2_6 and propene (Fig. S5†) is predicted by DFT to be about 8 × 10^3^ faster, compared to the formation of the SP molybdacyclobutane 3b from 1 and *trans*-2-butene (Fig. 5). This estimate also qualitatively agrees with the obtained kinetic parameters (*k*_2.1_/*k*_3_ ≈ 1750, Table S3†).

**Fig. 7:**
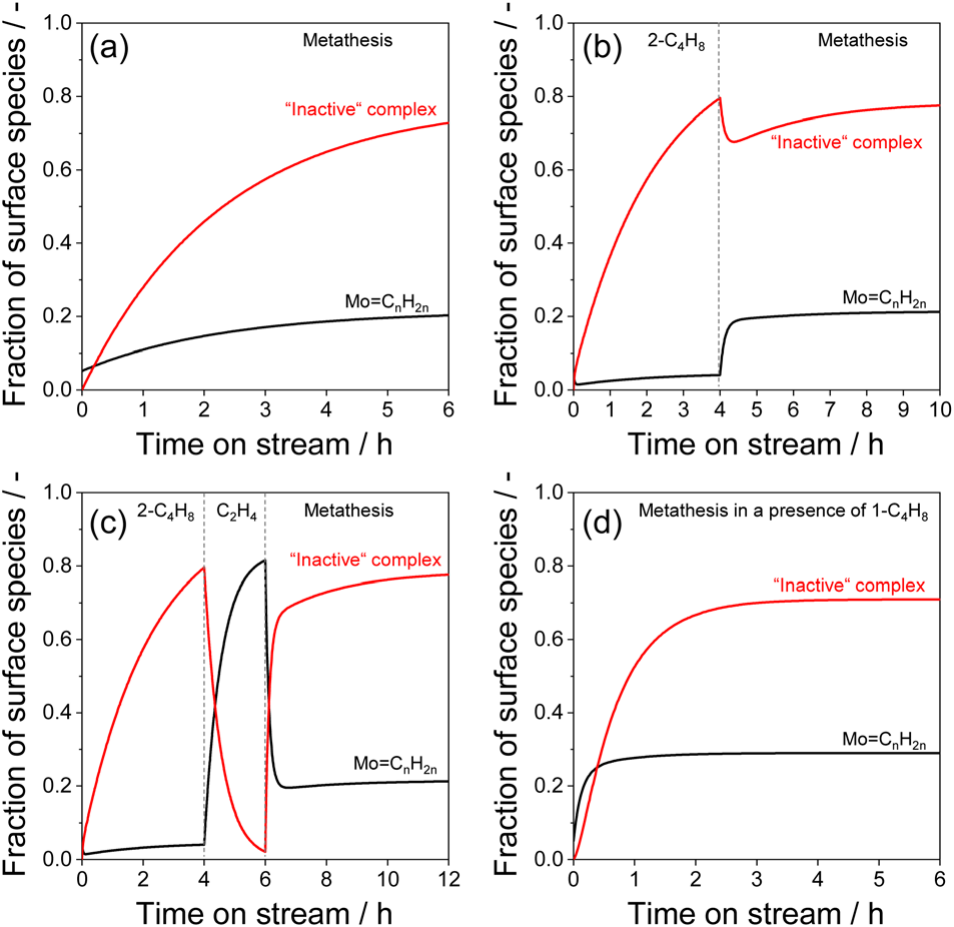
Time-on-stream changes of the fraction of surface species (Mo-carbenes and “inactive” complex) formed from MoO_*x*_ (a) during metathesis of ethylene with 2-butene over calcined Mo/P/SiO_2_, (b) during catalyst treatment in *trans*-2-C_4_H_8_ for 4 h followed by metathesis, (c) during catalyst treatment in *trans*-2-C_4_H_8_ for 4 h, then in C_2_H_4_ for 2 h followed by metathesis, (d) during metathesis in the presence of 5 vol% 1-C_4_H_8_. Fraction of surface species is determined with respect to the total amount of activated MoO_*x*_.

Using the developed kinetic model and the obtained kinetic parameters (reaction rate and equilibrium constants) from Table S3,† we calculated the temporal profiles of the “inactive” complex and active Mo-carbenes during the metathesis reaction and various catalyst treatments (Fig. 7 and S7†). On this basis, a major part of MoO_*x*_ species, which are able to form Mo-carbenes *in situ*, is present in the form of the “inactive” complex under steady-state conditions. Their fraction with respect to the total amount of activated MoO_*x*_ is around 73% (Fig. 7(a)). Thus, to increase the production of propene, it is required to transform the “inactive” species to Mo-carbenes. Given the developed kinetic model, this can be achieved by reducing the partial pressure of *trans*-2-C_4_H_8_ to shift the equilibrium of reaction 3 (Table S2†).

The model predicts an enhancement in the rate of propene formation by a factor of 1.5 when the ratio of C_2_H_4_/*trans*-2-C_4_H_8_ increases from 1 to 2 (Fig. S8(a)†). This is due to the decrease in the fraction of the “inactive” complex from 73% to 55% in favour of active Mo-carbenes (Fig. S8(b)†). To experimentally check this theoretical conclusion, we performed an additional experiment, in which untreated Mo/P/SiO_2_ was initially exposed to the reaction mixture C_2_H_4_/*trans*-2-C_4_H_8_/Ar = 10/10/2 with a total flow of 22 mL min^−1^ until a steady-state operation was achieved. Hereafter, this feed was replaced by a feed with more C_2_H_4_ and less *trans*-2-C_4_H_8_ (C_2_H_4_/*trans*-2-C_4_H_8_/Ar = 13.3/6.7/2). The total feed flow was not changed. As predicted by the model (Fig. S8(a)†), the rate of propene formation increased (Fig. S8(c)†). The activity decreased after switching back to the original feed after 2 h. Thus, the usage of reaction feeds with C_2_H_4_/*trans*-2-C_4_H_8_ ratios above the stoichiometric one is beneficial for increasing propene production.

## Conclusions

Herein, we provide novel insights into the formation and, particularly, transformation of Mo-carbenes in the metathesis of ethylene with 2-butene to propene over the Mo/P/SiO_2_ catalyst. There should be at least two types of MoO_*x*_ species participating in the formation of carbenes but differing in their reactivity in this process. Along with their involvement in the metathesis cycle, Mo-carbenes can be converted into an “inactive” complex formed with the participation of 2-butene. As this side reaction reduces the steady-state concentration of Mo-carbenes, the catalyst cannot achieve its highest possible activity. The fraction of the active and “inactive” species dynamically varies and can be controlled through catalyst treatments in certain olefins or changing the ethylene/2-butene ratio in the reaction mixture. The use of high ratios is beneficial in suppressing the formation of the undesired complexes and opens the possibility for increasing the steady-state metathesis activity of the catalyst.

## Data availability

The data supporting this article have been included as part of the Supplementary Information.†

## Author contributions

Conceptualization: T. O. and E. V. K.; investigation: T. O., J. H., and M. S.; formal analysis: A. F. and J. H.; writing – original draft: T. O., A. F., and J. H. All authors discussed the results and gave their approval for the final version.

## Conflicts of interest

There are no conflicts to declare.

## Supplementary Material

SC-OLF-D4SC06833E-s001
