# Supplementary material for: Time-resolved and theoretical analysis of Mo-carbene transformations in metathesis of ethylene with 2-butene
Source: Chem Sci. 2025 Jan 8;16(7):3141–9. doi: 10.1039/d4sc06833e (PMC11740092; doi:10.1039/d4sc06833e)
Supplement: SC-016-D4SC06833E-s001 [file SC-016-D4SC06833E-s001.pdf]

Supplementary Information (SI) for Chemical Science.
This journal is © The Royal Society of Chemistry 2025

Electronic Supplementary Information

Time-resolved and theoretical analysis of Mo-carbene transformations in metathesis of ethylene with 2-butene

Tatiana Otroshchenko,*[a] Aleksandr Fedorov,[a] Qiyang Zhang,[a] David Linke,[a] Jarosław Handzlik,[b] Mirjam Schröder,[a,c,d] Björn Corzilius,[a,c,d] and Evgenii V. Kondratenko*[a]

[a]*Leibniz-Institut für Katalyse e.V. an der Universität Rostock, Albert-Einstein-Str. 29a, D-18059 Rostock, Germany*

[b]*Cracow University of Technology, Faculty of Chemical Engineering and Technology, ul. Warszawska 24, 31-155 Kraków, Poland*

[c]*Institute of Chemistry, Albert-Einstein-Str. 27, University of Rostock, 18059 Rostock, Germany*

[d]*Department Life, Light & Matter, Albert-Einstein-Str. 25, University of Rostock, 18059 Rostock, Germany*

*To whom correspondence should be addressed. E-mail: tatiana.otroshchenko@catalysis.de (TO), evgenii.kondratenko@catalysis.de (E:V:K).

Catalyst synthesis

Commercial SiO_2 (Davisil, grade 646, Sigma-Aldrich) was calcined in flowing air at 500°C for 8 h prior to use as a support for catalyst preparation. The calcined support (specific surface area was 304 $m^2 \cdot g^{-1}$) was modified with phosphorous by incipient wetness impregnation with an aqueous solution of $NH_4H_2PO_4$ (99%, Merck) in the concentration required to achieve an atomic surface density of P of 1.5 $atoms \cdot nm^{-2}$. The support precursor was dried at 110°C overnight and finally calcined at 500°C in flowing air for 4 h (heating rate was 5 $K \cdot min^{-1}$). The promoted support material is denoted as P/SiO_2.

Incipient wetness impregnation was also applied to deposit molybdenum on the P/SiO_2 support using an aqueous solution of $(NH_4)_6Mo_7O_{24} \cdot 4H_2O$ (99%, Alfa Aesar). The impregnated solid material was dried at 110°C overnight and finally calcined at 500°C in flowing air for 8 h (heating rate was 5 $K \cdot min^{-1}$). For Mo, a nominal surface density of 1.5 $atoms \cdot nm^{-2}$ was chosen, which corresponds to about 7.3 wt % of Mo. The catalyst is abbreviated as $Mo/P/SiO_2$.

Catalyst characterization

UV-vis measurements were carried out using an Avantes spectrometer (AvaSpec-2048-USB2-RM) equipped with a high-temperature reflection UV-vis probe, an Ava-Light-DH-S-BAL deuterium-halogen light source and a CCD array detector. The samples were *in situ* calcined at 500°C in flowing air (15 mL·min^{-1}) for 1 h and cooled down to 50°C in the same flow. The UV-vis spectra were recorded at 50°C in the 200–800 nm range. $BaSO_4$ was used as a white standard for the calculation of the Kubelka-Munk function.

The *in situ* Raman spectra of the samples were collected on a Renishaw inVia Raman Microscope equipped with a reaction cell (Linkam) using a 442 nm laser and an X20 objective. Before collecting the spectra at 50°C, the samples were heated up to 500°C in He flow (10 mL·min^{-1}) at a heating rate of 10 K·min^{-1}. Hereafter, they were treated at 500°C in air flow (10 mL·min^{-1}) for 1 h and cooled down to 50°C in He flow (10 mL·min^{-1}).

In situ diffuse reflectance infrared Fourier transform spectroscopy (DRIFTS) measurements were performed using a Thermo Scientific Nicolet iS10 spectrometer equipped with a Harrick Praying Mantis and a high-temperature reaction chamber. Prior to collecting the spectra, each sample (SiO_2, Mo/SiO_2, P/SiO_2, and Mo/P/SiO_2) was heated in an N_2 flow (10 mL·min^{-1}, heating rate 10 K·min^{-1}) from room temperature to 450 °C, then calcined in an air flow (12 mL·min^{-1}) at 450 °C for 1 h and cooled down in an N_2 flow to 50 °C. The spectra were collected with a resolution of 4 cm^{-1} and an accumulation of 64 scans in the range of 400−4000 cm^{-1} under flowing N_2 (10 mL·min^{-1}).

XPS analysis was performed for Mo/P/SiO_2 sample with a ESCALAB220iXL (ThermoFisher Scientific) with monochromatic Al Kα radiation (1486.6 eV). For charge compensation low-energetic electrons of 10 eV were used (flood gun). The electron binding energy was referenced to adventitious carbon at 284.8 eV according to the advice of NIST. The peaks were fitted after subtracting a Shirley background with Gaussian-Lorentzian curves.

Solid state ^{31}P magic angle spinning (MAS) NMR experiments were performed at a 9.4 T Bruker AVANCE III HD spectrometer operating at 400.5 MHz proton frequency. The Bruker Ascend DNP widebore magnet was equipped with a 4 mm MAS WVT H/X/Y probe which was configured in X/H double mode with inserted range coil to reach the frequency of ^{31}P (162.1 MHz). All experiments were performed at room temperature. The ^{31}P chemical shift was referenced to 85% H_3PO_4 at 0.0 ppm. $(NH_4)_2HPO_4$ was used as a secondary reference with its signal set to 1.33 ppm. For the detection of ^{31}P signals at a MAS frequency of 12 kHz, direct excitation and rotor synchronized Hahn-echo experiments were performed. T_1 time constants were obtained with a saturation recovery experiment with Hahn-echo detection. In case of $Mo/P/SiO_2$, the sample was diluted with adamantane in a weight ratio of 1:3 because the pure undiluted powder did not allow for stable MAS rotation. To this end, adamantane was ground in a mortar, sample was added, rapidly ground and mixed thoroughly.

Catalytic tests

Catalytic tests were performed at 1.25 bar (abs.) in an in-house developed set-up equipped with 14 parallel operating continuous-flow fixed-bed quartz reactors. The catalyst (100 mg, sieve faction of 315–710 μm) was heated in flowing N_2 up to 500°C and calcined in air flow at 500°C for 3 h. Hereafter, it was cooled down in N_2 flow to 50°C and exposed to a flow (22 mL·min^{-1} per reactor) of C_2H_4/trans-2-C_4H_8/N_2=5/5/1. To investigate the effect of catalyst treatment with individual olefins on propene production, the same catalyst sample (unless otherwise specified) was regenerated in air flow at 500°C for 3 h, cooled down in N_2 flow to 50°C and treated in one of the following ways before performing the metathesis reaction: (i) exposed to a flow (22 mL·min^{-1} per reactor) of trans-2-C_4H_8/N_2=5/6 for 15 min, 1 h, 2 h, or 4 h; (ii) exposed to a flow of C_2H_4/N_2=5/6 for 4 h; (iii) exposed to a flow (22 mL·min^{-1} per reactor) of trans-2-C_4H_8/N_2=5/6 for 1 h, 2 h, or 4 h and then to a flow (22 mL·min^{-1} per reactor) of C_2H_4/N_2=5/6 for 1 h, 2 h, or 4 h.

The feed components and the reaction products were analyzed by an on-line gas chromatograph (Agilent 6890) equipped with AL/S capillary column (for hydrocarbons) connected to a flame ionization detector and PLOT/Q (for CO_2)/Molsieve 5 (for H_2, O_2, N_2, and CO) capillary column combination connected to a thermal conductivity detector. To calculate the rate of formation of olefins ($r(C_nH_{2n})$), equation 1 was used. The rate was determined at a degree of ethylene conversion of less than 15%.

$$r(C_nH_{2n}) = \frac{F_{feed} \times \frac{x_{N_2}^{in}}{x_{N_2}^{out}} \times x^{out}(C_nH_{2n})}{V_m \times m_{cat}} \quad (1)$$

Here, F_{feed} is the volumetric flow rate of the feed gas (mL·min^{-1}) under reference conditions (0°C, 1 atm), x_{N_2} with superscripts “in” or “out” stands for the molar fraction of N_2

at the reactor inlet or outlet, $x^{out}(C_nH_{2n})$ is the molar fraction of a certain olefin at the reactor outlet, V_m is the molar volume (22414 mL·mol^{-1}), and m_{cat} is catalyst weight (g).

Steady-state isotopic transient kinetic analysis

Steady-state isotopic transient kinetic analysis (SSITKA) experiments were performed in a tubular continuous-flow fixed-bed quartz reactor at 50°C using an in-house developed set-up. The catalyst (100 mg) was heated in Ar flow up to 500°C at a heating rate of 10 $K \cdot min^{-1}$, calcined in air flow at 500°C for 1 h, and then cooled down to 50°C° in Ar flow. Hereafter the catalyst was exposed to a flow of a mixture consisting of 1 vol% C_2H_4, 1 vol% trans-2-C_4H_8 and Ar (total flow was 5 $mL \cdot min^{-1}$). After about 20 min on stream, this feed was switched to a feed with isotopic labelled $^{13}C_2H_4$ (1 vol% $^{13}C_2H_4$, 1 vol% trans-2-C_4H_8, 5 vol% He in Ar, total flow 5 $mL \cdot min^{-1}$). The gas composition at the reactor outlet was monitored by an on-line mass spectrometer (Pfeiffer Vacuum Omni Star) and an on-line gas chromatograph (Agilent 7890A). Hereafter, the catalyst sample was regenerated in air flow at 500°C for 1 h and cooled down to 50°C in Ar flow. Then, the sample was treated in trans-2-C_4H_8 flow (trans-2-C_4H_8/Ar=10/12, total flow 22 $mL \cdot min^{-1}$) for 4 h and then in C_2H_4 flow (C_2H_4/Ar=10/12, total flow 22 $mL \cdot min^{-1}$) for 4 h. The treated sample was exposed to the non-labelled reaction feed and to the labelled one according to the procedure described above for the fresh sample.

Reaction kinetic modeling

The following set of equations were used for kinetic modelling of the metathesis reaction:

$$\frac{\partial C_i}{\partial t} = -f\frac{\partial C_i}{\partial x} + R_i \tag{2}$$

$$\frac{\partial \theta_i}{\partial t} = \frac{R_i}{N_0} \tag{3}$$

$$R_i = \rho_{cat}\sum_j \nu_j^i r_j \tag{4}$$

$$r_i = k_i N_0\left(\prod_j p^{n_j^+}_j \prod_j \theta^{m_j^+}_j - \frac{1}{K_i}\prod_j p^{n_j^-}_j \prod_j \theta^{m_j^-}_j\right) \tag{5}$$

$$\sum_i \theta_i = 1 \tag{6}$$

where C_i – the concentration of i-component in the gas phase (mol·cm^{-3}); θ_i – the coverage of surface i-intermediate (dimensionless); f – the linear velocity (cm·s^{-1}); t – time (s); x – the coordinate along the catalyst bed (cm); r_j – the rate of j-reaction (mol·g^{-1}·s^{-1}); ρ_{cat} – catalyst density (g·cm^{-3}); p_j – the pressure of i-component in the gas phase (bar); n_j^+, m_j^+, n_j^-, and m_j^- – the reaction orders with respect to the feed components and the reaction products, ν_j^i – the stoichiometric coefficient of i-compound for the j-reaction (negative for reagents and positive for products); k_i – the rate constant of i-reaction; K_i – the equilibrium constant of i-reaction; N_0 – the total amount of active sites (mol g^{-1}).

The system of partial differential equations was transformed into the system of ordinary differential equations by the finite difference method.[1] The backward difference was used to approximate the derivative of x. To solve a stiff system of ordinary differential equations, the numerical Adams/BDF method with automatic stiffness detection and switching was used.[2] The

search for kinetic parameters (rate and equilibrium constants) was carried out by minimizing the following loss function:

$$\frac{1}{n}\sum_{i=1}^{n}\left(C_i^{exp} - C_i^{pred}\right)^2 \qquad (7)$$

where C_i^{exp} – the experimental value of the concentration, C_i^{pred} – the calculated value of the concentration, n – the number of experimental points.

The kinetic parameter sensitivity analysis was performed to identify the significance of parameters applied in the kinetic model. For this, we varied the value of a selected kinetic parameter (while keeping the other parameters constant) to find an interval where the loss function increased by up to 10%. The same procedure was performed for all the parameters of the model. The absence of external heat transfer, external and internal diffusion limitations, the wall effect, and axial dispersion was checked by applying Mears and Weisz-Prater criteria (see supporting information). [3, 4]

The Nelder-Mead algorithm was used to minimize the loss function. [5] The Python programming language[6] (version 3.10) and its scientific libraries SciPy[7], NumPy[8] and LMFIT[9] were used for calculations.

For the kinetic modeling the following assumptions were made:

1. The total concentration of active sites N_0 that can be transformed into carbene species from olefins was assumed to be 10^{-6} mol g^{-1}. This value is slightly higher than the number of carbene species determined in the SSITKA experiment (Table S1), since SSITKA shows only the active sites participating into propene formation and does not take into account the sites transformed into "inactive" complex.

2. There are two types of MoO_x species (Mo* and Mo**) on the surface of the catalyst which differ in their ability (fast Mo* or slow Mo**) to form active carbenes through the

reaction with C_4 olefins. Although, there are several mechanisms proposed for carbene generation (pseudo-Wittig, allyl mechanism, 1,2-hydrogen shift mechanism, oxidative coupling, H-assisted mechanism) which imply the formation of carbenes with different composition ($Mo{=}C_nH_{2n}$, n = 1 – 4), we assume that catalyst activation leads to the formation of $Mo{=}C_2H_4$ carbenes. This assumption is reasonable because any of possible $Mo{=}C_nH_{2n}$ carbenes can form $Mo{=}C_2H_4$ carbenes through the reaction with trans-2-C_4H_8, once this olefin is passed over the catalyst. Accordingly, the formation of $Mo{=}C_2H_4$ carbenes can include several steps. To simplify the kinetic model, we assumed that the first elementary step of the activation mechanisms is the rate-determining stage, and thus, the rate of the overall process can be presented as:

$$r_a \approx r_1 = k_1 \cdot N \cdot p_{olefin} \approx r_2 \approx r_3 \ldots,$$

where r_a – the overall rate of the carbene formation, r_i – the rate for elementary step i; k_1 – the constant rate; N – the concentration of MoO_x species (which are able to be transformed into carbenes) on the catalyst surface, p_{olefin} – partial pressure of olefin. Due to the low concentration of active sites, the formation of side products and the consumption of reagents during activation were neglected.

In the case of MoO_x species which are quickly transformed into active carbenes (Mo*), the proposed model includes an additional assumption. Instead of including additional elementary steps describing the formation of carbene species, it is assumed that the non-treated catalyst has already a certain amount of carbenes on the surface (θ_0 – the initial fraction of carbenes $Mo{=}C_2H_4$ that is an additional parameter of the kinetic model). As before, the formation of side products and the consumption of reagents during activation were neglected.

3. Because the partial pressure of olefins in the reaction feed is high, the decomposition of carbenes was not considered. All reactions and the values of kinetic parameters are presented in Tables S2 and S3.

Computational methods

A cluster model of *syn*-Mo(VI) ethylidene species on SiO_2 was obtained previously,[10] based on the periodic model of the dioxo Mo(VI) species on amorphous silica.[11] The dangling bonds are saturated with hydrogens replacing the removed Si atoms to form hydroxyl groups at the cluster periphery. Structures of other Mo species were calculated during the investigation of the reaction pathways.

Geometry optimization was carried out using the B3PW91 hybrid functional[12] combined with the def2-SVP basis set.[13] Vibrational frequencies were calculated in the harmonic oscillator approximation to confirm local minima or transition state structures, and to determine thermal corrections to the Gibbs energy. The rotational and translational contributions to the Gibbs energy are not included for the cluster models, only for gas-phase compounds. The transition states were additionally verified by the IRC method.[14-16] Single-point energy calculations were performed for the optimized structures with the M06 hybrid functional[17] and the def2-TZVPP basis set.[13] The total Gibbs energy for each system is estimated as the sum of the M06 single point energy and the thermal correction, including the zero point energy. Reaction pathways are presented in terms of relative Gibbs energies at T = 323.15 K. All DFT calculations were done with the Gaussian 16 software.[18]

Diffusion and heat transfer limitations

External diffusion limitation is negligible if:

$$\frac{r_{obs} \cdot \rho_b \cdot R_{catalyst} \cdot n}{k_d \cdot C} < 0.15$$

Obtained value: ≈ $1.4 \cdot 10^{-3} < 0.15$

Internal diffusion limitation is negligible if:

$$\frac{r_{obs} \cdot \rho_p \cdot R^2_{catalyst}}{D_e \cdot C} < 1$$

Obtained value: ≈ $1.5 \cdot 10^{-2} < 1$

External heat transfer limitation is negligible if:

$$\frac{r_{obs} \cdot \rho_b \cdot R_{catalyst} \cdot E_a \cdot \Delta H}{k_g \cdot R \cdot T^2} < 0.15$$

Obtained value: ≈ $1.2 \cdot 10^{-4} < 0.15$

Axial dispersion is negligible if:

$$\frac{20 \cdot n \cdot d_{reactor}}{Pe_{ax} \cdot L_{bed}} < 1$$

Obtained value: ≈ 0.35 <1

The wall effect is negligible if:

$$\frac{d_{reactor}}{2 \cdot R_{catalyst}} > 10$$

Obtained value: ≈ 7.8 <10

Thus, there is a small wall effect. Nevertheless, since catalytic tests were performed with the same catalyst bed, the wall effect is equal for all measurements and was neglected.

The following equations and parameters values were used for estimation:

a. $Sh = \frac{2 \cdot k_d \cdot R_{catalyst}}{D} = 2 + 0.552 \cdot Re^{1/2} \cdot Sc^{1/3}$

b. $Re = \frac{2 \cdot u_{feed} \cdot R_{catalyst} \cdot \rho_{feed}}{\mu}$

c. $Sc = \frac{\mu}{\rho_{feed} \cdot D}$

d. $D_e = \frac{D \cdot \varepsilon \cdot \delta}{\tau}$

e. $$\frac{1}{Pe_{ax}} = \frac{0.3}{Re{\cdot}Sc} + \frac{0.5}{1 + \frac{3.8}{Re{\cdot}Sc}}$$

The values of the parameters used for the validation of diffusion and heat transfer limitations are listed below.

	**Parameter**	**Dimension**	**Value**
r_{obs}	measured reaction rate	mol·g^{-1}·min^{-1}	$4{\cdot}10^{-4}$
ρ_b	bulk catalyst density	kg·m^{-3}	468
ρ_p	particle density	kg·m^{-3}	780
$R_{catalyst}$	catalyst particle radius	mm	0.256
n	reaction order	-	2
k_d	mass transfer coefficient [a]	m·s^{-1}	0.053
C	bulk concentration of reactant	kmol·m^{-3}	21.15
Sh	Sherwood number [a]	-	2.705
Re	Reynolds number [b]	-	2.5
Sc	Schmidt number [c]	-	0.53
D	diffusion coefficient	m^2·s^{-1}	10^{-5}
D_e	diffusion coefficient in porous [d]	m^2·s^{-1}	$1.06{\cdot}10^{-6}$
u_{feed}	gas feed velocity	m·s^{-1}	0.0257
ρ_{feed}	gas feed density	kg·m^{-3}	1.895
μ	dynamic viscosity	kg·m^{-1}·s^{-1}	10^{-5}
ε	porosity	-	0.4
δ	constrictivity	-	0.8
τ	tortuosity	-	3.0
E_a	activation energy	kJ·mol^{-1}	100
ΔH	reaction heat	kJ·mol^{-1}	25.65
k_g	heat transport coefficient	kJ·m^{-2}·s^{-1}·K^{-1}	2.0
R	gas constant	J·mol^{-1}·K^{-1}	8.314
T	temperature	K	323.15
$d_{reactor}$	reactor diameter	mm	4
L_{bed}	catalyst bed length	cm	1.7
Pe_{ax}	Peclet number [e]	-	2.81

Tables

Table S1 Rate of propene formation, surface residence time of intermediates, concentration of surface intermediates, turnover frequency, fraction of Mo transformed into carbenes with respect to the total amount of Mo in the sample. The samples are denoted as follows: $Mo/P/SiO_2$_cal – calcined $Mo/P/SiO_2$, $Mo/P/SiO_2$_C_4-C_2 – $Mo/P/SiO_2$ sequentially treated in 2-C_4H_8 at 50°C for 4 h and in C_2H_4 at 50°C for 4 h.

Sample	$r(C_3H_6)$, mol·g·s^{-1}	τ_P, s	N_P, mol·g^{-1}	TOF, s^{-1}	Fraction of Mo[#], %
$Mo/P/SiO_2$_cal	$7.3\cdot10^{-9}$	13.1	$9.6\cdot10^{-8}$	$7.6\cdot10^{-2}$	$1.3\cdot10^{-2}$
$Mo/P/SiO_2$_C_4-C_2	$7.12\cdot10^{-8}$	9.8	$7.0\cdot10^{-7}$	$1.0\cdot10^{-1}$	$9.2\cdot10^{-2}$

[#] Fraction of Mo participating in carbene formation was calculated with respect to the total amount of Mo in the sample based on the assumption that one carbene site is formed from one Mo atom.

Table S2 Reactions considered for kinetic modeling.

#	reaction	kinetic expression
	Carbene formation	
1.1	trans-2-C_4H_8 + Mo** →→ Mo=C_2H_4 + products	$k_{1.1} \cdot N_0 \cdot \theta \cdot p_{2-C_4H_8}$
1.2	1-C_4H_8 + Mo** →→ Mo=C_2H_4 + products	$k_{1.2} \cdot N_0 \cdot \theta \cdot p_{1-C_4H_8}$
	Metathesis	
2.1	C_2H_4 + Mo=C_2H_4 → Mo=CH_2 + C_3H_6	$k_{2.1} \cdot N_0 \cdot \theta_2 \cdot p_{C_2H_4}$
2.2	trans-2-C_4H_8 + Mo=CH_2 → Mo=C_2H_4 + C_3H_6	$k_{2.2} \cdot N_0 \cdot \theta_1 \cdot p_{2-C_4H_8}$
2.3	1-C_4H_8 + Mo=C_2H_4 → Mo=C_3H_6 + C_3H_6	$k_{2.3} \cdot N_0 \cdot \theta_2 \cdot p_{1-C_4H_8}$
2.4	trans-2-C_4H_8 + Mo=C_3H_6 → Mo=C_2H_4 + C_5H_{10}	$k_{2.4} \cdot N_0 \cdot \theta_3 \cdot p_{2-C_4H_8}$
	"Inactive" complex formation	
3	trans-2-C_4H_8 + Mo=C_2H_4 ⇄ Mo=C_6H_{12}	$k_3 \cdot N_0 \cdot (\theta_2 \cdot p_{2-C_4H_8} - 1/K_3 \cdot \theta_6)$

p_i – the partial pressure of the compound i. N_0 - the total concentration of active sites; θ - fraction of surface species with respect to the total amount of active sites. For each surface species, its fraction is provided below.

Surface species	Fraction [value at t = 0 s is shown in brackets*]
Mo**	θ [$1 - \theta_0$]
CH_2 ‖ Mo	θ_1 [0]
C_2H_4 ‖ Mo	θ_2 [θ_0]
C_3H_6 ‖ Mo	θ_3 [0]
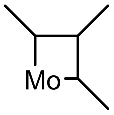	θ_6 [0]

*initial condition for the integration of the system of differential equations.

Table S3 The values of the estimated parameters for the proposed kinetic model and their intervals based on parameter sensitivity analysis.

parameter	value	Interval	unit
θ_0	0.05	0 – 0.12	-
$k_{1.1}$	0.00046	0.00043-0.00054	$bar^{-1}\ s^{-1}$
$k_{1.2}$	0.017	0.014-0.020	$bar^{-1}\ s^{-1}$
$k_{2.1}$	67.9	65.7-69.8	$bar^{-1}\ s^{-1}$
$k_{2.2}$	15.0	13.6-16.2	$bar^{-1}\ s^{-1}$
$k_{2.3}$	2460	2310-2540	$bar^{-1}\ s^{-1}$
$k_{2.4}$	210	130-640	$bar^{-1}\ s^{-1}$
k_3	0.039	0.032-0.044	$bar^{-1}\ s^{-1}$
K_3	35.6	35.3-37.2	bar^{-1}

Table S4 The comparison of the values of the key kinetic parameters estimated by DFT and data-driven kinetic modelling.

	kinetic modelling	DFT
**$k_{2.1}$/ $k_{2.2}$**	4.5	13
**$k_{2.1}$/ k_3**	1750	$8.1 \cdot 10^3$
**$k_{2.2}$/ k_3**	388	623
**K_3**	35.6	8.1

Figures

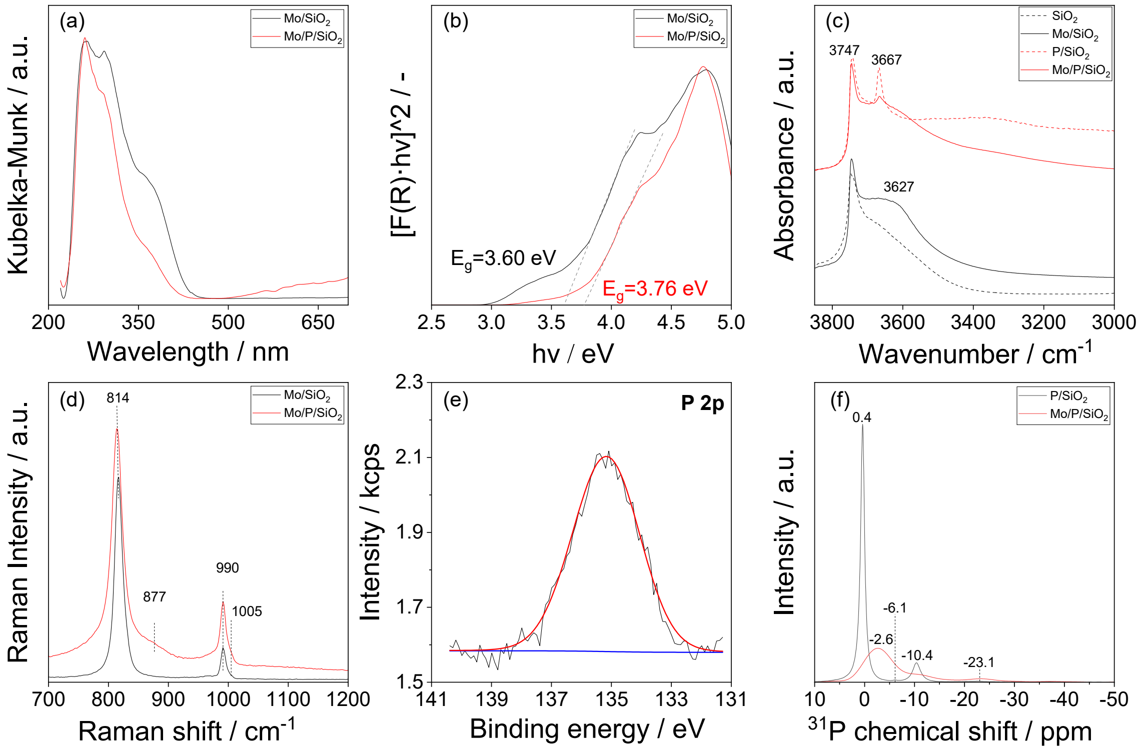

Figure S1 (a) UV-vis spectra and (b) the corresponding Tauc plots; (c) DRIFT spectra of support materials and the corresponding Mo-containing samples; (d) Raman spectra of dehydrated Mo/P/SiO_2 and reference Mo/SiO_2; (e) XPS of P 2p region of Mo/P/SiO_2; (f) ^{31}P MAS-NMR of the rotating P/SiO_2 and Mo/P/SiO_2 diluted with adamantane. The spectra are normalized to the number of scans.

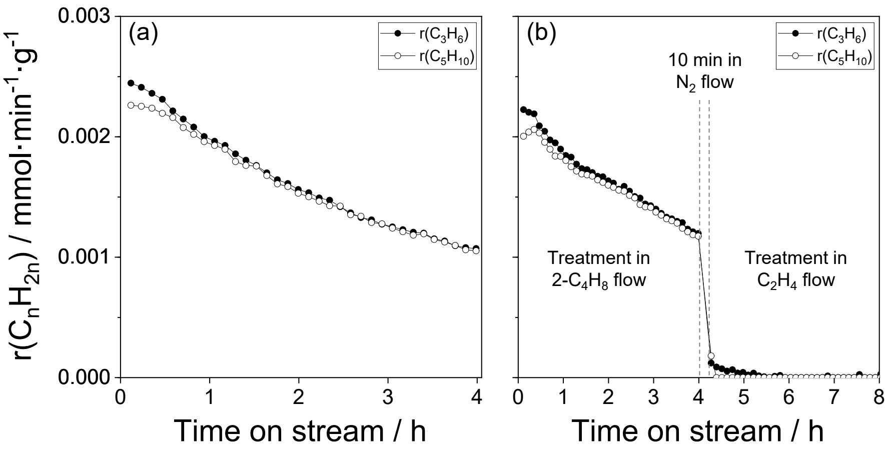

Figure S2 Temporal changes in the rates of formation of propene and pentenes during treatment of $Mo/P/SiO_2$ in (a) trans-2-C_4H_8 for 4 h at 50°C; (b) trans-2-C_4H_8 for 4 h at 50°C and then in C_2H_4 for 4 h at 50°C.

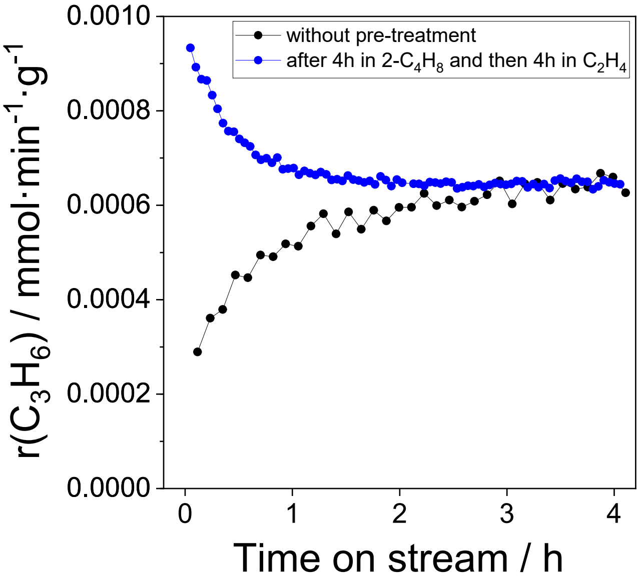

Figure S3 Temporal changes of the rate of propene formation determined over calcined Mo/SiO_2 (marked in black) and Mo/SiO_2 after treatment in trans-2-C_4H_8 for 4 h followed by treatment in C_2H_4 for 4h (marked in blue). Treatments and metathesis reaction were carried out at 50°C.

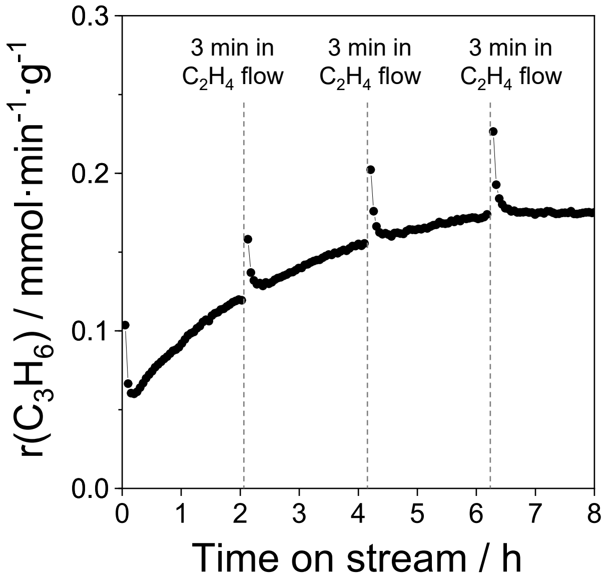

Figure S4 Temporal changes in the rate of propene formation over regenerated Mo/P/SiO_2 treated in trans-2-C_4H_8 at 50°C for 1 h and then in C_2H_4 for 30 min (dash lines indicate *in situ* treatment in C_2H_4 flow for 3 min after certain periods of time on stream). Unlike other experiments that used the same catalyst sample after regeneration, this experiment was performed with another catalyst sample, so its metathesis performance is slightly different due to the inhomogeneity of the catalyst surface.

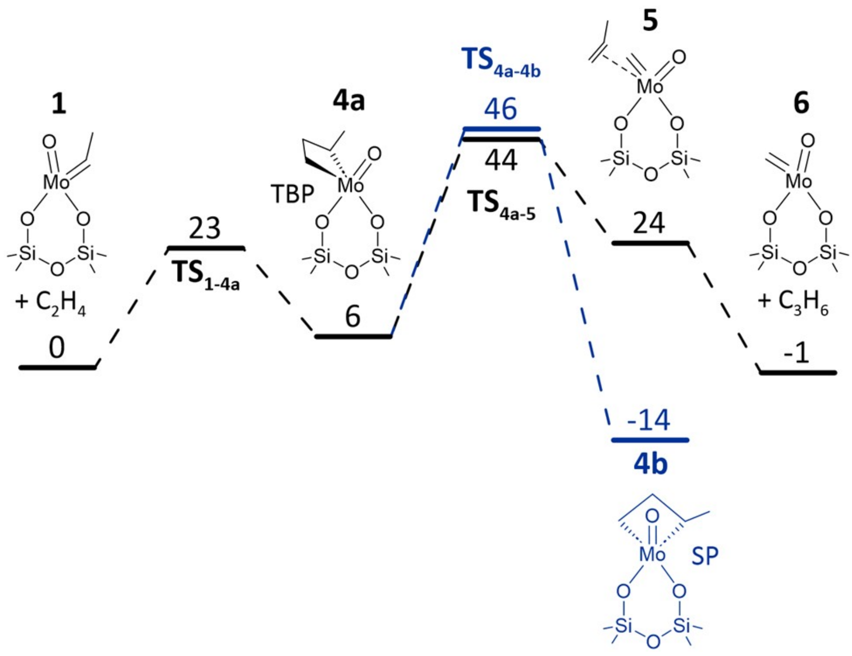

Figure S5 Gibbs energy profile (kJ mol^{-1}) at T = 323 K for a reaction between *syn*-Mo(VI) ethylidene species and ethylene.

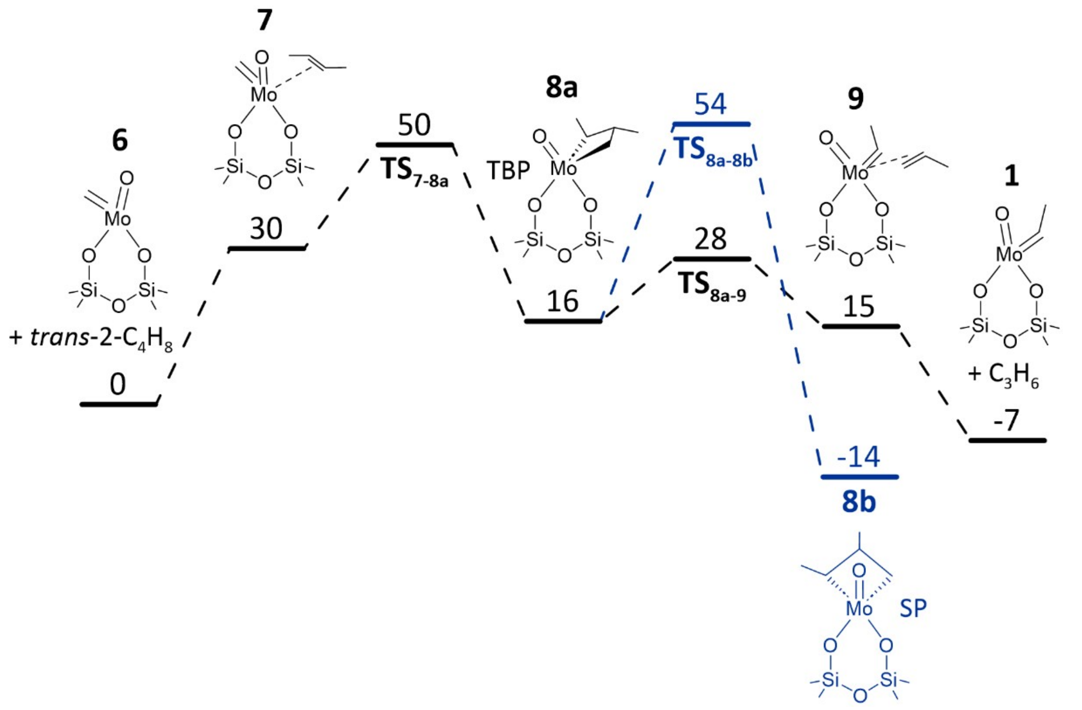

Figure S6 Gibbs energy profile (kJ mol^{-1}) at T = 323 K for a reaction between Mo(VI) methylidene species and *trans*-2-butene.

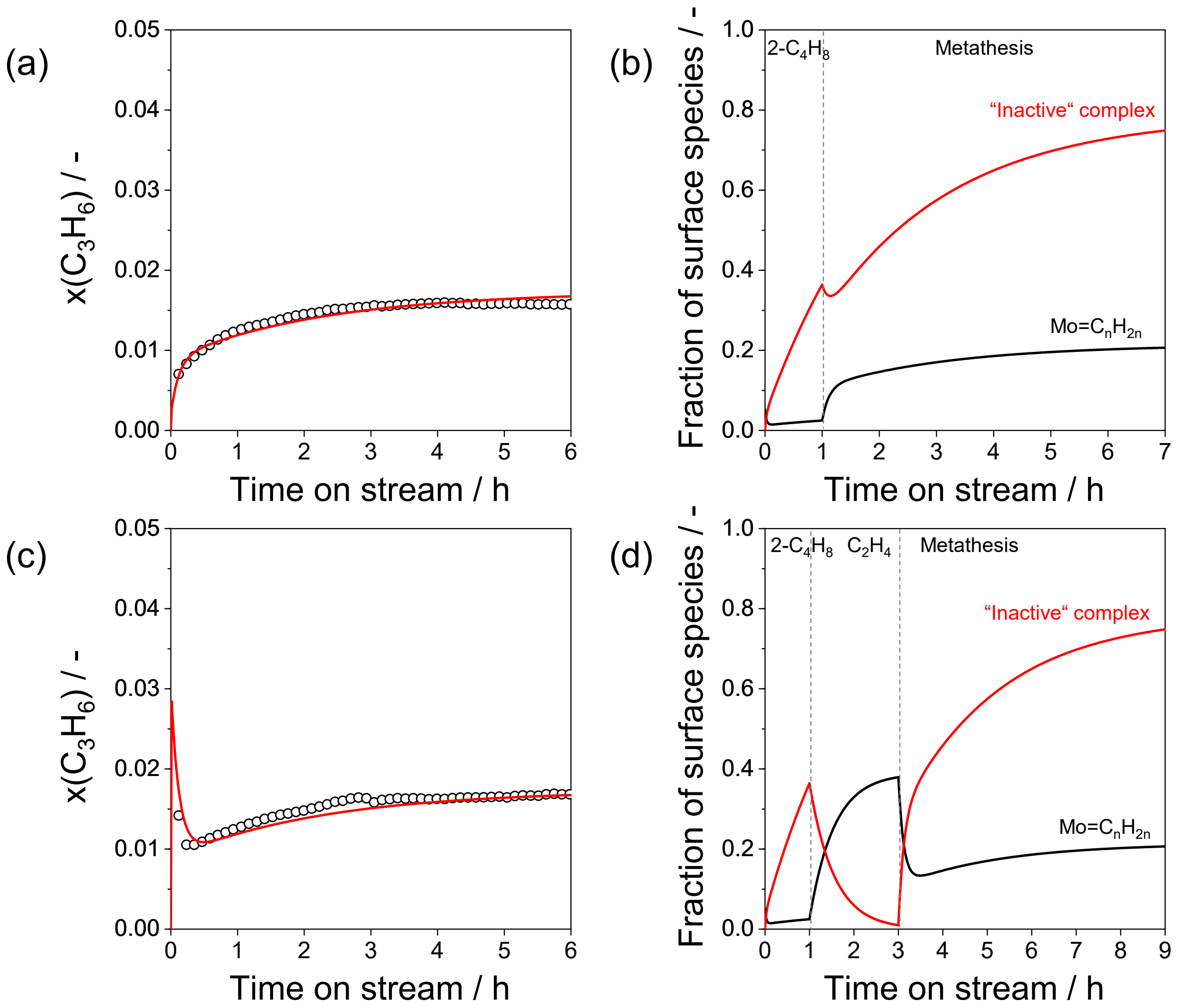

Figure S7 Experimental data and fitting results ((a) and (c)) as well as time-on-stream changes of the fractions of surface species (Mo-carbenes and “inactive” complex) formed from MoO_x ((b) and (d)) during metathesis over $Mo/P/SiO_2$ treated in trans-2-C_4H_8 for 1 h ((a), (b)) and treated in trans-2-C_4H_8 for 1 h and then in C_2H_4 for 2 h ((c), (d)). The changes of the fractions of surface species also include the data obtained for catalyst pre-treatment. Fraction of surface species is determined with respect to the total amount of activated MoO_x.

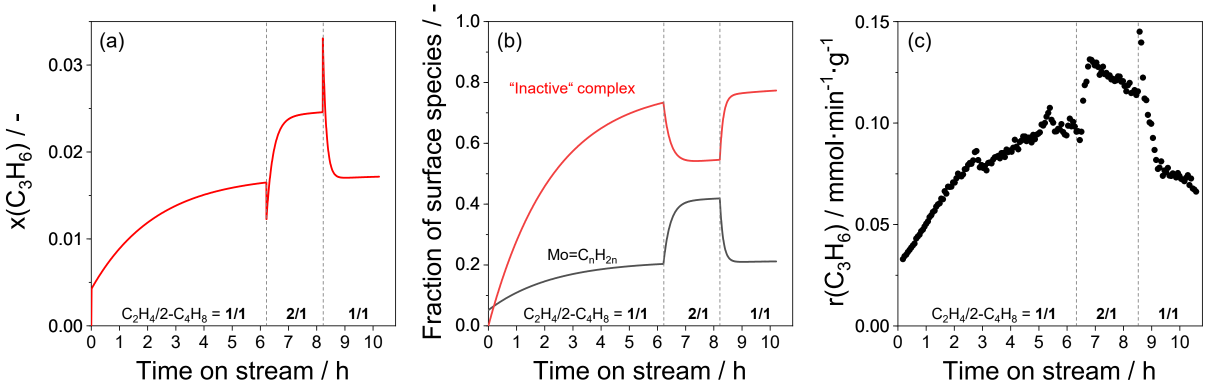

Figure S8 Prediction for temporal change of (a) propene molar fraction and (b) the fractions of surface species (Mo-carbenes and "inactive" complex); (c) experimentally obtained temporal changes in the rate of propene formation over calcined Mo/P/SiO_2. The reaction mixture C_2H_4/trans-2-C_4H_8/Ar=10/10/2 was applied for the first 6.3 h on stream, then the mixture was changed to C_2H_4/trans-2-C_4H_8/Ar=13.3/6.7/2 (next 2 h on stream), and again to C_2H_4/trans-2-C_4H_8/Ar=10/10/2. Total flow and the reaction temperature were 22 mL·min^{-1} and 50°C respectively. Fraction of surface species is determined with respect to the total amount of activated MoO_x.

Due to the technical restrictions, catalytic data shown in Figure S8 (c) were obtained in a SSITKA setup, which differs from the setup (BERTA) applied for all other catalytic experiments. Moreover, different catalyst sample was used. Accordingly, the model prediction (Figure S8 (a) and (b)) obtained on the basis of the BERTA experimental data might not perfectly fit the data obtained in SSITKA setup. Nevertheless, it demonstrates the same tendency when C_2H_4/trans-2-C_4H_8 ratio was changed.

Cartesian coordinates (Å) of the structures

1

O	-1.68495400	0.02429000	2.34873200
O	-3.47607400	3.09109800	-1.47051600
O	-0.03666900	-0.50866900	4.46418300
O	-3.90098800	-3.76749100	-0.29191100
O	-4.39957000	0.91074500	-0.15987500
O	0.29581800	-1.79387000	2.21643700
O	-1.45420800	-3.10566300	0.69626600
O	-2.47869700	-1.92511500	-1.48892200
O	0.87885900	0.85991300	2.31687100
O	0.91570600	-4.26130100	1.33518100
O	-2.44024100	-0.80586600	-3.96186300
O	0.48429300	2.89608900	0.60749700
O	-2.25844900	0.72615300	-1.82524600
O	0.87437800	-2.27615600	-0.38124500
O	-1.22959100	3.99030900	2.27260000
O	0.40461200	0.33415300	-0.21232500
O	-0.20846300	-0.92242400	-2.45740200
O	2.74875500	1.43262700	0.38837300
O	2.40222100	-0.51720200	-1.75153800
O	-2.06369800	2.12120600	0.49604300
O	-3.50223700	-1.40673600	0.96810100
O	-1.42207700	4.59957000	-0.37573900
O	4.95324800	1.23362500	-1.79711900
H	-0.17183700	0.28664200	4.98970400
Si	-0.13337100	-0.33989400	2.83975300
Si	-2.83234600	-2.55874500	-0.02017100
Si	0.14797900	-2.86427900	0.97325400
Si	-3.07722100	1.65195500	-0.74227700
H	-4.26059200	0.02692300	0.25019200
H	-2.18453700	-1.56484100	-4.49607800
H	0.70901500	-4.66356000	2.18463900
Si	-1.85532300	-0.73892700	-2.43712000
Si	1.15829900	1.41712000	0.78840800
H	-1.90254400	4.66526500	2.40956100
Si	0.88400100	-0.87196100	-1.23752100
H	-4.36380200	-4.11303300	0.47783200
Si	-1.06309800	3.45279700	0.72929700
H	-3.96173100	3.04172500	-2.30155500
H	-2.14765500	4.34186900	-0.97539800
H	-1.81981400	0.84222800	1.82378900
H	-2.88288300	-0.99880400	1.62267100
Mo	3.80533100	0.45406100	-0.87768800
C	4.83003800	-0.77295400	0.09238200
H	4.14457600	-1.35283000	0.74259100
C	6.27516400	-1.12625000	0.16516700
H	6.88548600	-0.50840000	-0.50872400
H	6.65332800	-0.99823700	1.19480000
H	6.42719400	-2.18748400	-0.09981100

2

O	-2.61514000	0.13209500	2.11318600
O	-3.45842200	3.11720100	-2.09791600
O	-1.47696100	-0.38039700	4.54108700
O	-4.32819000	-3.67037100	-0.85930600
O	-4.68930300	0.98947200	-0.96538600
O	-0.71512600	-1.74732500	2.45465500
O	-2.13178100	-3.06806500	0.62477800

O	-2.61541500	-1.90526900	-1.75180600
O	-0.07536800	0.89902700	2.61358200
O	0.00853600	-4.25014700	1.79409700
O	-2.00617500	-0.89693200	-4.19145400
O	-0.02797800	2.86909200	0.79588600
O	-2.24867200	0.72909700	-2.13628800
O	0.39242000	-2.32880600	0.04252900
O	-2.01916400	4.09421500	2.00255000
O	-0.11359300	0.28499400	0.07035900
O	-0.15615300	-0.99183300	-2.23429900
O	2.15322600	1.27658200	1.05760100
O	2.21624500	-0.60258900	-0.92771200
O	-2.52809500	2.17967600	0.15310300
O	-4.12747900	-1.29501700	0.41521800
O	-1.60206200	4.58764400	-0.64267400
O	4.54620300	1.85419800	-0.24645400
H	-1.67644400	0.44176100	5.00066600
Si	-1.21079300	-0.25653600	2.92892400
Si	-3.29794700	-2.48958400	-0.38210400
Si	-0.61779600	-2.84527200	1.23161700
Si	-3.25540000	1.69278100	-1.26357600
H	-4.66270600	0.12397300	-0.49709000
H	-1.74586300	-1.72775100	-4.60194900
H	-0.35559500	-4.57377400	2.62410200
Si	-1.75957400	-0.77184700	-2.57712400
Si	0.53961100	1.36820900	1.14702400
H	-2.69148700	4.78166000	1.95414200
Si	0.62546100	-0.91865500	-0.78495200
H	-4.95038400	-3.98338900	-0.19523100
Si	-1.53599200	3.47803300	0.55660900
H	-3.72700900	3.04217900	-3.02040000
H	-2.20471300	4.33205400	-1.36617200
H	-2.59784700	0.92650000	1.53787400
H	-3.64671900	-0.87835100	1.17451100
Mo	3.58678500	0.50322400	-0.02163600
C	4.65943400	-0.15503500	-2.24574900
H	3.73242300	-0.46386700	-2.74219200
C	5.22890500	-1.05239900	-1.37673700
C	4.56299200	-0.48543200	1.26082100
H	4.02130800	-1.41825400	1.51091700
C	5.82077300	-0.24573200	2.01513600
H	6.33828800	0.66612900	1.68715600
H	5.58740700	-0.14065600	3.09037200
H	6.50909700	-1.10590300	1.93108800
H	6.22430800	-0.80785100	-0.98444200
C	5.37069900	1.02648300	-2.83450200
H	5.70840900	0.75469400	-3.85036100
H	4.71553400	1.90328200	-2.93457600
H	6.24994600	1.32040800	-2.24565800
C	4.78800100	-2.48096300	-1.23593500
H	5.04548200	-2.90700500	-0.25620300
H	3.70754700	-2.59098400	-1.39952200
H	5.31079800	-3.08392300	-1.99906500

3a

O	-2.65734300	0.09867300	2.10254000
O	-3.40066100	3.19559400	-2.05316400
O	-1.55105800	-0.49477000	4.52782600
O	-4.36230100	-3.61064000	-1.00407900
O	-4.66873500	1.05672600	-0.98592400

O	-0.77855500	-1.81009300	2.41092200
O	-2.18531800	-3.06842900	0.53170700
O	-2.62061400	-1.84110000	-1.82438800
O	-0.11756400	0.82407600	2.65027400
O	-0.08243300	-4.30761800	1.70917700
O	-1.96334200	-0.77248300	-4.22546600
O	-0.00529900	2.83190000	0.86534600
O	-2.21236800	0.79644300	-2.12801200
O	0.35454800	-2.35464800	-0.00026200
O	-1.98363100	4.07650000	2.06994400
O	-0.11576100	0.25826000	0.09335000
O	-0.14144500	-0.94880500	-2.24763300
O	2.14281700	1.22003100	1.15575900
O	2.21418500	-0.59839500	-0.87949700
O	-2.50878400	2.19845700	0.18636000
O	-4.15659000	-1.26946200	0.33431700
O	-1.52720600	4.60068300	-0.56512200
O	4.86609000	1.27525300	0.90394300
H	-1.74843400	0.31466700	5.01026700
Si	-1.26872400	-0.33225500	2.92300100
Si	-3.32768400	-2.45411800	-0.48018600
Si	-0.67643800	-2.88284000	1.16394200
Si	-3.22377700	1.74918100	-1.25068000
H	-4.65874000	0.17658700	-0.54420100
H	-1.70358900	-1.59229900	-4.65790900
H	-0.42079100	-4.61134900	2.55738300
Si	-1.73581200	-0.69846800	-2.60515300
Si	0.50986200	1.31205500	1.20090800
H	-2.64174400	4.77815000	2.03067500
Si	0.63145000	-0.93046400	-0.79096200
H	-4.97970600	-3.95591000	-0.35163300
Si	-1.50201900	3.47387000	0.61878600
H	-3.67391400	3.14665000	-2.97607500
H	-2.13134300	4.37091600	-1.29649100
H	-2.62504900	0.90886900	1.55126000
H	-3.68637900	-0.88183500	1.11410900
Mo	3.58908800	0.47040400	0.15322000
C	4.47337200	0.73916800	-1.67959400
H	3.73359700	0.56588000	-2.47157400
C	5.10526600	-0.66262400	-1.22373800
C	4.65644700	-1.28234900	0.18469000
H	3.97748600	-2.12771400	0.01489500
C	5.76637400	-1.59489900	1.15342900
H	6.39850400	-0.71634700	1.34701600
H	5.34519700	-1.93246000	2.11275900
H	6.40367700	-2.41338600	0.77438600
H	6.17060700	-0.43427900	-1.08069000
C	5.45729800	1.83050000	-2.01025700
H	6.04402800	1.57670100	-2.91091300
H	4.92224900	2.76774600	-2.22718100
H	6.15174200	2.01831800	-1.17870400
C	4.86736100	-1.69785800	-2.32325600
H	5.37208700	-2.64557700	-2.08194100
H	3.79039300	-1.89067800	-2.42919400
H	5.25665700	-1.33789600	-3.28768300

3b

O	-1.94005000	0.09548100	2.40185000
O	-4.40727800	2.51408800	-1.50777900
O	-0.09950200	-0.01390500	4.41863000

O	-3.77093300	-4.19262600	0.25942300
O	-4.92113600	0.35869400	0.04719600
O	0.26250700	-1.44802900	2.26811700
O	-1.38301500	-3.13152200	1.01625000
O	-2.68575400	-2.29574500	-1.18079200
O	0.47997500	1.25424500	2.09786000
O	1.17529900	-3.87382800	1.54672200
O	-2.99101200	-1.42954100	-3.73675200
O	-0.33123600	3.05254300	0.27005000
O	-2.89359400	0.31378200	-1.76275600
O	0.70962800	-2.09206400	-0.32224000
O	-2.07357800	4.05424200	1.96134800
O	-0.11840000	0.43279800	-0.31903000
O	-0.66696800	-1.07931800	-2.41853300
O	2.08558300	1.89317300	-0.04922300
O	1.89611500	-0.25271700	-1.91382300
O	-2.74408300	1.93165000	0.41498700
O	-3.62294500	-1.69574000	1.29193300
O	-2.51941700	4.38286000	-0.70589700
O	4.43671500	1.52702000	-2.04583700
H	-0.33465900	0.79292200	4.88844300
Si	-0.32424900	-0.01148100	2.79812900
Si	-2.86305000	-2.83520800	0.35576700
Si	0.18130600	-2.64228900	1.13693800
Si	-3.76018300	1.21629500	-0.69719900
H	-4.62916400	-0.45416300	0.51871500
H	-2.66215900	-2.19227200	-4.22346400
H	1.12759100	-4.18879000	2.45486500
Si	-2.31852000	-1.13073800	-2.27759300
Si	0.55486700	1.69998900	0.50860900
H	-2.82498200	4.64199000	2.09234800
Si	0.47151300	-0.77580000	-1.28056100
H	-4.12242900	-4.53537300	1.08713700
Si	-1.93121000	3.40333800	0.45983100
H	-4.93172700	2.32261400	-2.29343300
H	-3.23829900	3.97499600	-1.22480300
H	-2.22535900	0.83016200	1.81713500
H	-3.02675300	-1.14792500	1.85978500
Mo	3.27365600	0.80498200	-1.09488200
C	4.27162800	-1.09163500	-0.71899600
H	3.49987800	-1.69338800	-0.20615600
C	5.27758500	-0.56374000	0.29133300
C	4.40293700	0.59185200	0.75139300
H	3.64616900	0.22586500	1.46963900
C	5.02815100	1.88557300	1.23433700
H	5.75094200	2.28515900	0.50589900
H	4.27224900	2.66054500	1.42956700
H	5.57305300	1.71369400	2.18086900
H	6.14344300	-0.15016400	-0.25787700
C	4.74320400	-1.78319100	-1.98300100
H	5.22202000	-2.74708700	-1.73017300
H	3.91049700	-2.00513100	-2.66690400
H	5.48452400	-1.17784200	-2.52763200
C	5.77567600	-1.53509900	1.35736800
H	6.43866300	-1.03340300	2.08081700
H	4.93149500	-1.97001000	1.91767600
H	6.34435900	-2.36600700	0.90942800

4a

O	-2.21772400	0.07487100	2.24841500

O	-3.41840500	3.15671900	-1.81401400
O	-0.85706700	-0.50544700	4.54413400
O	-4.18612800	-3.65835900	-0.67270500
O	-4.54786400	1.00603000	-0.62201300
O	-0.29330700	-1.80982500	2.35538200
O	-1.86986000	-3.08991900	0.63297000
O	-2.55532600	-1.87195800	-1.66758700
O	0.35646800	0.83144000	2.52918900
O	0.36019300	-4.30026400	1.58628100
O	-2.16133000	-0.79481700	-4.12279100
O	0.25715900	2.84092700	0.74356400
O	-2.21786700	0.77014000	-2.00817000
O	0.59342300	-2.34427700	-0.15719100
O	-1.60367000	4.05669300	2.14766500
O	0.10571500	0.26398100	-0.01538000
O	-0.14517400	-0.94503400	-2.34140400
O	2.44573400	1.25917600	0.81355900
O	2.33415700	-0.56815800	-1.21410400
O	-2.29209300	2.17296400	0.32317300
O	-3.86978800	-1.31260700	0.63591800
O	-1.42800600	4.59084800	-0.51873400
O	5.12761700	1.36409100	0.24438600
H	-1.01902800	0.30005200	5.04598000
Si	-0.74632100	-0.33937900	2.91905600
Si	-3.11606500	-2.48997800	-0.25836300
Si	-0.30643500	-2.88450600	1.10593500
Si	-3.14511200	1.71356200	-1.03341100
H	-4.48238600	0.12637900	-0.18433600
H	-1.92116500	-1.60417700	-4.58522900
H	0.09684400	-4.62167300	2.45433800
Si	-1.77139800	-0.71748500	-2.53424700
Si	0.82113000	1.32851000	1.02372300
H	-2.26826600	4.75266400	2.17840300
Si	0.77134300	-0.91824600	-0.97172100
H	-4.73363600	-4.00560900	0.03844900
Si	-1.26613000	3.46324700	0.65294200
H	-3.78286600	3.10422000	-2.70467000
H	-2.09741900	4.35164700	-1.18762900
H	-2.25401800	0.88505800	1.69746900
H	-3.32754400	-0.92002100	1.36456000
Mo	3.78292900	0.52715500	-0.33090700
C	4.46172600	0.73241300	-2.26313800
H	3.70041100	0.61893200	-3.03946400
H	5.19262100	1.52133700	-2.46032600
C	5.11463000	-0.63323900	-1.83417700
C	4.85209900	-1.21842000	-0.35448700
H	4.72377700	-1.39893800	-2.51348500
H	4.16657200	-2.07207700	-0.41583800
C	6.07832600	-1.48723000	0.47683500
H	6.72545600	-0.60146300	0.54910500
H	5.78098300	-1.77833600	1.49588600
H	6.66482900	-2.32402500	0.05901000
H	6.20208600	-0.51350800	-1.90348900

4b

O	-1.71044700	0.15957400	2.40357000
O	-3.84416500	2.97626400	-1.44124100
O	0.09820000	-0.23360600	4.41398700
O	-4.05227500	-3.80589700	0.14003000
O	-4.62888600	0.85741300	0.04956200

O	0.28544500	-1.64090700	2.22384100
O	-1.55414300	-3.06211900	0.91974200
O	-2.74528600	-2.01489900	-1.24959300
O	0.83520200	1.01729300	2.12869000
O	0.88307100	-4.14326100	1.43586400
O	-2.92118000	-1.04682900	-3.78122700
O	0.25799700	2.95126600	0.34948200
O	-2.61244900	0.61393100	-1.75799200
O	0.66387500	-2.26412000	-0.38259700
O	-1.34793400	4.11291500	2.07288100
O	0.14763000	0.34043800	-0.30953100
O	-0.57951100	-1.03484100	-2.44827800
O	2.51238900	1.50958600	-0.00180500
O	2.06172200	-0.53310600	-1.92990900
O	-2.27546400	2.13705300	0.46580700
O	-3.60357900	-1.37448300	1.24085200
O	-1.74476700	4.57322000	-0.58292200
O	4.75156400	0.94935000	-2.08289500
H	-0.03967900	0.58143400	4.90762000
Si	-0.12159900	-0.15884100	2.79475900
Si	-2.98680600	-2.57177600	0.27176300
Si	0.05920600	-2.78319100	1.05875800
Si	-3.36715800	1.58528500	-0.66816500
H	-4.44254200	0.00233500	0.49964400
H	-2.68554800	-1.82836000	-4.29164000
H	0.80688700	-4.46371900	2.34009900
Si	-2.22479500	-0.87769700	-2.31275100
Si	0.96517300	1.49257000	0.55281300
H	-2.02027200	4.78553500	2.22386100
Si	0.58643100	-0.90507600	-1.30586500
H	-4.44144000	-4.12908900	0.95869600
Si	-1.28651100	3.49419600	0.55256700
H	-4.38512100	2.87454800	-2.23245000
H	-2.50606300	4.27219100	-1.11431000
H	-1.90153000	0.93938200	1.83950000
H	-2.94690600	-0.92370400	1.82644600
Mo	3.55458400	0.33730300	-1.09899800
C	4.31666700	-1.66167800	-0.79965100
H	3.51546900	-2.25753500	-0.33651400
H	4.56786900	-2.09043700	-1.78324900
C	5.43088000	-1.28899800	0.15660800
C	4.73675300	-0.04828800	0.69153800
H	5.70588700	-2.04988000	0.91105600
H	3.97364700	-0.33297000	1.43750000
C	5.53894300	1.14780900	1.16048600
H	6.27146000	1.46951300	0.40376500
H	4.89606200	2.00670200	1.40500900
H	6.10223800	0.88748300	2.07603100
H	6.34633200	-1.01367400	-0.39189700

5

O	-2.35594700	0.09045300	2.15988300
O	-3.36274500	3.04034100	-2.04315100
O	-1.14183600	-0.39044000	4.55996000
O	-4.04618800	-3.77361100	-0.75665300
O	-4.50950700	0.89720500	-0.85506200
O	-0.40263000	-1.74157300	2.45479100
O	-1.81855000	-3.09533200	0.64577100
O	-2.42547300	-1.96569400	-1.72167500
O	0.17070100	0.92216600	2.59094000

O	0.34869300	-4.23370700	1.80616300
O	-1.88353300	-0.93972400	-4.16919200
O	0.17668000	2.86752300	0.74127700
O	-2.10270000	0.67672300	-2.10283600
O	0.67441800	-2.31603600	0.02288800
O	-1.79305000	4.10860300	1.96543300
O	0.09321300	0.27600600	0.05176900
O	0.00859400	-1.01150700	-2.25047700
O	2.36702300	1.27943800	0.99330500
O	2.40283200	-0.53181300	-1.04454800
O	-2.33482600	2.14264500	0.17927600
O	-3.86739500	-1.37984900	0.49476100
O	-1.43984500	4.53195900	-0.70505100
O	5.01053300	0.43118400	0.91844700
H	-1.32560100	0.42870100	5.03126400
Si	-0.92375000	-0.26422900	2.94210400
Si	-3.03623200	-2.56038500	-0.32174000
Si	-0.30392100	-2.84535000	1.23699400
Si	-3.10176200	1.62677700	-1.20631600
H	-4.45257500	0.02799500	-0.39569800
H	-1.64425300	-1.77459000	-4.58442100
H	0.15380000	-4.44577800	2.72451300
Si	-1.60600600	-0.81659700	-2.56037200
Si	0.75049700	1.37395600	1.10886500
H	-2.47305000	4.78866900	1.92067600
Si	0.82930500	-0.91237300	-0.82785200
H	-4.60219100	-4.13609400	-0.05981600
Si	-1.34427500	3.45769100	0.52443900
H	-3.69305900	2.95530700	-2.94449000
H	-2.07902200	4.26755700	-1.39336800
H	-2.36855200	0.88741000	1.58898300
H	-3.37930300	-0.95223300	1.24266400
Mo	3.75575700	0.59171000	-0.17183600
C	4.25407700	2.07924700	-1.21591300
H	3.59643300	2.40077100	-2.03873500
H	5.16252300	2.67572500	-1.06900300
C	5.11559300	-0.45111500	-2.15283700
C	5.13748400	-1.43254500	-1.20893500
H	4.27651800	-0.36464600	-2.84635100
H	4.25698300	-2.08119700	-1.13471800
C	6.33370300	-1.82163400	-0.40414700
H	7.13928800	-1.07741100	-0.46955400
H	6.08132500	-1.97037700	0.65530400
H	6.71569900	-2.78421400	-0.78661500
H	6.00641200	0.14636000	-2.36637000

6

O	-1.67998200	0.28715300	2.28925500
O	-2.01991000	3.91785700	-1.45647000
O	-0.50160200	-0.82138400	4.49865400
O	-4.71748800	-2.47742000	-0.80312700
O	-3.70946700	2.10235300	-0.37848400
O	-0.36110600	-2.05378300	2.19547400
O	-2.31126600	-2.67376100	0.44764600
O	-2.67573100	-1.14286400	-1.73657200
O	1.00308000	0.27269900	2.53728700
O	-0.67758800	-4.56822600	1.26436900
O	-2.00448400	-0.00377400	-4.10628700
O	1.44919100	2.38761000	0.92956500
O	-1.57119000	1.30098000	-1.85847300

O	0.25838500	-2.60568300	-0.34637500
O	0.01910000	3.94271100	2.49108100
O	0.65506400	0.02258100	-0.06351700
O	-0.10827300	-0.90129600	-2.41340400
O	3.13821600	0.26822300	0.81497800
O	2.40881100	-1.41617800	-1.44805100
O	-1.18760100	2.48031600	0.55263000
O	-3.71575500	-0.41488400	0.65176200
O	0.30368900	4.62972300	-0.13477700
O	5.34764400	-1.38386700	-0.52903500
H	-0.43042900	-0.04827900	5.06791600
Si	-0.38115800	-0.57009200	2.88835500
Si	-3.35931100	-1.68877400	-0.35023300
Si	-0.76859000	-2.98050200	0.89634100
Si	-2.16938000	2.40111700	-0.79379300
H	-3.90195200	1.20512900	-0.02066300
H	-2.01426600	-0.79546700	-4.65405700
H	0.12131600	-4.87437300	1.70501000
Si	-1.60238300	-0.19187400	-2.53328000
Si	1.58995500	0.76596500	1.08173500
H	-0.41805600	4.79250300	2.60894400
Si	0.80748700	-1.25698900	-1.10153500
H	-5.26751300	-2.83382800	-0.09819700
Si	0.15490900	3.41246800	0.94323900
H	-2.44695400	4.07053900	-2.30688500
H	-0.41534600	4.64762100	-0.79491800
H	-1.49904800	1.12855800	1.81984400
H	-3.07509300	-0.26567200	1.38904500
Mo	3.93511900	-0.52989500	-0.72291500
C	4.41620700	0.83471500	-1.90624800
H	3.62911900	1.54906300	-2.19603200
H	5.41072300	1.00040000	-2.33672600

7

O	-1.99333400	0.25347300	2.40298200
O	-2.93646500	3.79216500	-1.36346400
O	-0.53334400	-0.76289000	4.47082200
O	-5.12636500	-2.73677800	-0.36663800
O	-4.38722300	1.88188700	-0.11161000
O	-0.53698900	-2.01246900	2.17376100
O	-2.60177700	-2.76742700	0.63732400
O	-3.27963600	-1.28547400	-1.50980000
O	0.70145400	0.39963700	2.36244000
O	-0.78688800	-4.55392000	1.28520900
O	-2.90040200	-0.18316800	-3.95839500
O	0.84149200	2.50182700	0.69296300
O	-2.35361500	1.21430400	-1.79604500
O	-0.11971400	-2.56478400	-0.41465800
O	-0.51368000	4.01227700	2.35857800
O	-0.00184700	0.07917000	-0.14083900
O	-0.80343600	-0.91810100	-2.43900200
O	2.58992900	0.48619100	0.36828100
O	1.81265900	-1.14379200	-1.66854200
O	-1.82713600	2.44498600	0.57408600
O	-4.12124700	-0.59859500	0.96722700
O	-0.53215800	4.65251400	-0.29120400
O	4.40135300	-1.97890100	-1.06969100
H	-0.54882100	0.02760600	5.01990500
Si	-0.58545300	-0.52293900	2.85194100
Si	-3.78058100	-1.85807900	-0.05957300

Si	-1.00233600	-2.97508800	0.91878800
Si	-2.91752900	2.27629300	-0.67704300
H	-4.47561900	0.98427800	0.28510500
H	-2.84013600	-0.97662200	-4.50023500
H	0.11787000	-4.84647400	1.43242600
Si	-2.33715900	-0.30088000	-2.42600600
Si	1.07187700	0.88111700	0.82408100
H	-1.03443800	4.80868700	2.50585200
Si	0.25734700	-1.16629500	-1.20529000
H	-5.56947800	-3.12157300	0.39620800
Si	-0.50231800	3.44840900	0.81427000
H	-3.40713600	3.88055600	-2.19989300
H	-1.31429900	4.61182200	-0.87441000
H	-1.90930200	1.07928900	1.88215600
H	-3.42222400	-0.39974700	1.63706300
Mo	3.53813000	-0.55168800	-0.99834400
C	5.32625400	-0.10947000	0.87596100
H	4.58708500	0.12851600	1.64974100
C	5.62646500	0.87414000	-0.02689300
C	4.20512200	0.45254100	-2.44699600
H	3.79593500	1.45771200	-2.63313100
C	6.14818400	-1.34371100	1.08920800
H	6.79921800	-1.56372100	0.23204100
H	6.78681900	-1.18546400	1.97624200
H	5.52917500	-2.22955200	1.28756400
H	4.97689100	0.11310000	-3.14832100
C	5.17131700	2.29933600	0.08333400
H	4.20201000	2.38391700	0.59211800
H	5.10648500	2.79462700	-0.89528500
H	5.91821900	2.85515400	0.67714900
H	6.44934400	0.67917300	-0.72619300

8a

O	-2.08180600	0.31141900	2.35808600
O	-3.08383600	3.62807800	-1.56120900
O	-0.64871200	-0.54719900	4.51364200
O	-4.97910100	-2.95685500	-0.33514100
O	-4.46840300	1.70312800	-0.25614000
O	-0.53855500	-1.90257200	2.28817100
O	-2.47300800	-2.83083900	0.71205400
O	-3.17093600	-1.47072700	-1.50257400
O	0.60447800	0.56652400	2.38716600
O	-0.43357400	-4.45594600	1.45282100
O	-2.80753200	-0.45179200	-3.99151800
O	0.71272000	2.59291900	0.62927000
O	-2.38144600	1.06344200	-1.88033500
O	0.00619500	-2.53817900	-0.29479100
O	-0.75651900	4.11141800	2.19002400
O	-0.00254200	0.11378600	-0.12246200
O	-0.70522300	-1.00400400	-2.40346100
O	2.54529800	0.60270700	0.43571600
O	1.89237200	-1.10361700	-1.58614400
O	-1.94986800	2.41030500	0.44726700
O	-4.11271700	-0.72663100	0.92652400
O	-0.72999800	4.63232100	-0.48500400
O	4.55754200	-1.07655000	-2.17441600
H	-0.67665400	0.26819100	5.02464400
Si	-0.65615300	-0.37964700	2.88379900
Si	-3.68051900	-2.00430800	-0.04040700
Si	-0.86448400	-2.93205100	1.04400300

Si	-3.00799700	2.14441600	-0.81305600
H	-4.52499100	0.81728100	0.17007500
H	-2.67571000	-1.25586600	-4.50428200
H	-0.66885700	-4.74384700	2.34036600
Si	-2.27194100	-0.47177700	-2.44580600
Si	1.02526100	0.99231800	0.84197300
H	-1.30733000	4.89581800	2.28177900
Si	0.32049000	-1.15420200	-1.12869900
H	-5.50133600	-3.21522300	0.43070300
Si	-0.67275600	3.47999100	0.67374900
H	-3.53514000	3.65966600	-2.41225800
H	-1.49849700	4.53688400	-1.07911200
H	-2.01582900	1.11691600	1.80239400
H	-3.43779000	-0.45056500	1.59538900
Mo	3.56145900	-0.37544300	-1.01057300
C	4.92835900	-0.77055300	0.45395500
H	4.44949700	-0.67692500	1.43744500
C	5.41044100	0.70029600	-0.03642100
C	4.58346300	1.39944700	-1.18419300
H	4.04194000	2.28512700	-0.83693600
C	5.97535700	-1.85090100	0.39786000
H	6.40153900	-1.95315300	-0.61053300
H	6.79431100	-1.64778500	1.11037300
H	5.53237600	-2.81775800	0.68196500
H	5.15818100	1.55814600	-2.10080800
C	5.48907300	1.61298300	1.18744900
H	4.48197600	1.78250400	1.59367600
H	5.92425000	2.58566800	0.91370800
H	6.11565900	1.16389900	1.97257200
H	6.39570000	0.49653400	-0.47715900

8b

O	-1.88433800	-0.06463700	2.38336700
O	-4.40771900	2.37623800	-1.47669800
O	-0.08082500	-0.15601200	4.43544300
O	-3.43073500	-4.35258300	0.02749800
O	-4.83082700	0.13233400	-0.02314700
O	0.40147100	-1.47813600	2.23723300
O	-1.12035200	-3.19689400	0.88044700
O	-2.42190000	-2.34103300	-1.30905400
O	0.47327200	1.23673600	2.18128600
O	1.46619200	-3.81512900	1.43549900
O	-2.72897200	-1.38580800	-3.83310200
O	-0.40520500	3.06502700	0.41462000
O	-2.76792500	0.27557900	-1.78749500
O	0.93470100	-1.98486000	-0.36726000
O	-2.23700100	3.89606100	2.10389700
O	-0.03195100	0.48967900	-0.27993800
O	-0.45392900	-0.96093900	-2.45181400
O	2.07779500	2.05761200	0.09185900
O	2.05061400	-0.02048100	-1.85220900
O	-2.75271000	1.80631200	0.45690300
O	-3.44069700	-1.89649200	1.16390600
O	-2.64379600	4.31117200	-0.55777700
O	4.48710300	1.90564300	-1.82278300
H	-0.35549000	0.62206100	4.93160700
Si	-0.27365600	-0.09855600	2.81214000
Si	-2.60110500	-2.95341500	0.19989900
Si	0.41048700	-2.62595700	1.05661400
Si	-3.70515300	1.08348300	-0.70547200

H	-4.50332600	-0.68174400	0.42204200
H	-2.35521600	-2.11392800	-4.34000100
H	1.42249600	-4.16895000	2.32944900
Si	-2.10297300	-1.11155300	-2.34886900
Si	0.55235000	1.75638300	0.61519000
H	-3.02070300	4.43855800	2.24067800
Si	0.63987100	-0.64531400	-1.27505400
H	-3.77906200	-4.74920500	0.83218200
Si	-2.02612700	3.31820300	0.58090400
H	-4.90747100	2.18936100	-2.27936600
H	-3.32834900	3.88663200	-1.10869000
H	-2.19943200	0.67791100	1.82448500
H	-2.88691300	-1.34069900	1.76609300
Mo	3.34675500	1.06256600	-0.94892900
C	4.46095800	-0.78000900	-0.62194400
H	3.71022600	-1.45771300	-0.17667000
C	5.37622500	-0.23746500	0.46781600
C	4.39280300	0.82568400	0.92206800
H	3.66198700	0.44726200	1.65565800
C	5.03456200	-1.36753300	-1.89600100
H	5.75736000	-0.68595500	-2.37095600
H	5.56456300	-2.31152500	-1.67158400
H	4.25056600	-1.60478600	-2.63091400
H	4.76634000	1.80837300	1.25387100
C	5.87711900	-1.22581200	1.51490300
H	5.03382500	-1.73965500	2.00537200
H	6.46097200	-0.71426500	2.29734200
H	6.52425300	-1.99615500	1.06515600
H	6.23979700	0.25650900	-0.01215900

9

O	-2.02934700	0.27009700	2.37354800
O	-3.44398900	3.37395700	-1.58597800
O	-0.45561200	-0.39882400	4.49566900
O	-4.68730200	-3.32897400	-0.12570200
O	-4.60535100	1.37817600	-0.17333000
O	-0.27788200	-1.79000500	2.29584200
O	-2.16642600	-2.92860000	0.80932200
O	-3.07807000	-1.71157100	-1.41002400
O	0.61845800	0.77838900	2.30562800
O	0.03625500	-4.34327600	1.50890700
O	-2.87636400	-0.72213700	-3.92739000
O	0.50437700	2.74729200	0.48441400
O	-2.53157700	0.87118000	-1.85932000
O	0.25465600	-2.43849300	-0.28519000
O	-1.03687200	4.17537200	2.06170600
O	-0.02240800	0.19486000	-0.17281000
O	-0.68856000	-1.04990000	-2.39792200
O	2.50036000	0.91154200	0.33215900
O	1.93544300	-0.83382100	-1.68117200
O	-2.13709300	2.32441400	0.41242600
O	-3.99764400	-0.98959900	1.03694000
O	-1.15815300	4.61861700	-0.62331600
O	4.45617300	-0.01458400	-2.44550400
H	-0.57844000	0.41907100	4.98838500
Si	-0.52900900	-0.27332100	2.86305300
Si	-3.47696000	-2.24566900	0.08481100
Si	-0.54416300	-2.87314200	1.08113300
Si	-3.21040400	1.92611200	-0.80024800
H	-4.57065200	0.49813500	0.26654900

H	-2.63315000	-1.49499800	-4.44726800
H	-0.26870900	-4.69354400	2.35169000
Si	-2.29357500	-0.65964800	-2.39918500
Si	0.94733600	1.18379100	0.73251300
H	-1.64865700	4.91344400	2.15165000
Si	0.41166800	-1.05403500	-1.17385900
H	-5.18365800	-3.56813500	0.66320000
Si	-0.95361500	3.50924800	0.56008000
H	-3.91290100	3.33525800	-2.42720300
H	-1.93280000	4.43340600	-1.18768500
H	-2.05322900	1.06314800	1.79652000
H	-3.33010200	-0.63371500	1.67461500
Mo	3.59732200	-0.06445400	-1.00821600
C	4.47043900	-1.47429000	-0.08628300
H	3.99669900	-1.63932000	0.89973700
C	5.57721300	0.92664100	0.36700800
C	4.97579900	1.84602500	-0.46174700
H	4.24812600	2.55460400	-0.05860500
C	5.55415200	-2.42269600	-0.44995600
H	6.02713300	-2.17175100	-1.40924300
H	6.32581500	-2.47508000	0.33875100
H	5.12779700	-3.43915400	-0.53223700
H	5.39714500	2.05502500	-1.44690800
C	5.36466900	0.88332800	1.85083100
H	4.34076000	1.17902300	2.11758100
H	6.06008300	1.59978800	2.32190800
H	5.57894800	-0.10575500	2.27893500
H	6.44615200	0.38395300	-0.02179000

$TS_{2\text{-}3a}$

O	-2.60114100	0.07456800	2.13437100
O	-3.52480500	3.13009700	-2.01505000
O	-1.42479100	-0.47827500	4.53453900
O	-4.31056800	-3.68250500	-0.90347500
O	-4.72023200	0.96782000	-0.91079700
O	-0.67828900	-1.79375400	2.40941200
O	-2.10699100	-3.09557000	0.57612900
O	-2.61742600	-1.88569300	-1.77160800
O	-0.06109800	0.85452900	2.61609900
O	0.06175900	-4.27638000	1.69265200
O	-2.04808600	-0.82553300	-4.19957800
O	-0.05902200	2.85933200	0.83127000
O	-2.28848700	0.75791300	-2.11225000
O	0.40259700	-2.31984500	-0.02751700
O	-2.04376100	4.04689700	2.08415400
O	-0.13138100	0.28646700	0.06287500
O	-0.17402200	-0.93563400	-2.26739400
O	2.14012300	1.29211000	1.03698700
O	2.20613700	-0.55064400	-0.95159900
O	-2.56070100	2.16042500	0.20787000
O	-4.11747600	-1.33427900	0.42003900
O	-1.66402400	4.58969300	-0.55749800
O	4.68711000	1.81079300	0.18135000
H	-1.63025200	0.33155900	5.01297100
Si	-1.18226100	-0.31920100	2.92180900
Si	-3.28463800	-2.50445600	-0.41009800
Si	-0.58594900	-2.86754900	1.16395900
Si	-3.29713000	1.69181200	-1.21081700
H	-4.67886500	0.09431000	-0.45860800
H	-1.75765500	-1.63438100	-4.63307300

H	-0.30485500	-4.63039200	2.50905300
Si	-1.78308500	-0.72809100	-2.58603700
Si	0.52117500	1.35629600	1.14826900
H	-2.72300900	4.72869500	2.05740400
Si	0.62387400	-0.88814800	-0.82433500
H	-4.92591000	-4.01235700	-0.24117200
Si	-1.57544600	3.46006900	0.62137300
H	-3.80027300	3.06913600	-2.93656500
H	-2.27403800	4.34159200	-1.27762200
H	-2.59941300	0.87908300	1.57329300
H	-3.63351500	-0.92929300	1.18322600
Mo	3.60131100	0.54553400	-0.00893800
C	4.58787200	0.20383100	-2.02461400
H	3.75145800	-0.10755600	-2.65992100
C	5.18432100	-0.84525200	-1.26985500
C	4.58995200	-0.83545000	0.89240500
H	4.01596000	-1.77729200	0.92489000
C	5.83112600	-0.81214400	1.71190900
H	6.40090200	0.11794600	1.58071900
H	5.54116300	-0.88714000	2.77504200
H	6.47968700	-1.68054400	1.50300600
H	6.21356200	-0.65625600	-0.94100400
C	5.39278000	1.37032600	-2.54234500
H	5.83747600	1.10376600	-3.51734000
H	4.76708500	2.26070400	-2.70247200
H	6.20484700	1.64962200	-1.85725000
C	4.83940800	-2.28701200	-1.54922900
H	5.23184300	-2.97603500	-0.78754900
H	3.75198500	-2.42192400	-1.63144200
H	5.28906000	-2.57195900	-2.51539800

$TS_{3a\text{-}3b}$

O	-2.42728500	0.14277300	2.27824800
O	-3.86190100	2.90417100	-1.94567500
O	-0.98772100	-0.23696600	4.56351000
O	-4.27812800	-3.85413200	-0.36144500
O	-4.86600900	0.78365700	-0.59381800
O	-0.41656500	-1.65284900	2.44761600
O	-1.95213700	-3.09595200	0.82272100
O	-2.76693100	-2.05929700	-1.52234400
O	0.12396800	1.01420400	2.43500700
O	0.35254500	-4.14422300	1.78903900
O	-2.45865100	-1.11955100	-4.04738400
O	-0.12584500	2.91070100	0.54609100
O	-2.56444300	0.56557600	-2.02851100
O	0.46802700	-2.26845200	-0.04437800
O	-2.01072500	4.12428500	1.91691300
O	-0.23242600	0.28810600	-0.05827400
O	-0.41253400	-1.07312300	-2.29980200
O	2.11036700	1.37692500	0.58106900
O	2.04981500	-0.46712700	-1.27459600
O	-2.64951800	2.10598900	0.22201600
O	-4.03935000	-1.41974900	0.78609200
O	-1.91938500	4.50796100	-0.77741600
O	4.14263900	1.95040000	-1.27970000
H	-1.23389300	0.57844000	5.01230200
Si	-0.92221400	-0.17027000	2.92811100
Si	-3.25404500	-2.61174900	-0.06061500
Si	-0.39341800	-2.78858500	1.25247200
Si	-3.50674200	1.52538000	-1.08523900

H	-4.74707600	-0.06204000	-0.10447100
H	-2.09608900	-1.89188500	-4.49305900
H	0.03792300	-4.50073800	2.62570200
Si	-2.05003300	-0.92694600	-2.47378300
Si	0.49932100	1.43361500	0.87967700
H	-2.70899100	4.78724000	1.92047500
Si	0.52107800	-0.88827100	-0.95516500
H	-4.81562700	-4.14940200	0.38011000
Si	-1.67771200	3.45846300	0.45121900
H	-4.21196900	2.77712000	-2.83468500
H	-2.58469600	4.19501200	-1.41949200
H	-2.51135100	0.90735000	1.66993600
H	-3.50033000	-0.95808800	1.47522000
Mo	3.52819000	0.61616800	-0.49711400
C	4.65506000	-0.84520900	-1.50049400
H	4.17750600	-1.82960400	-1.39061000
C	5.60390400	-0.56657100	-0.28978400
C	4.82891400	0.04535300	0.96220300
H	4.37766500	-0.78013800	1.54184300
C	5.61040400	1.00361000	1.83304300
H	6.01051100	1.84942100	1.25446300
H	4.96111500	1.41449200	2.62052000
H	6.45760500	0.49888100	2.33025700
H	6.36215800	0.17230400	-0.59497200
C	5.21296000	-0.63340600	-2.88581300
H	5.98673100	-1.38343000	-3.13292400
H	4.41170400	-0.73899200	-3.63328100
H	5.65480200	0.36756600	-3.00216100
C	6.28375000	-1.87521000	0.14081700
H	6.96203400	-1.69670800	0.98933800
H	5.53763100	-2.62482000	0.44399200
H	6.86899800	-2.29197800	-0.69283500

$TS_{1\text{-}4a}$

O	-2.21107600	0.12906900	2.23998400
O	-3.16466400	3.36033900	-1.77996100
O	-0.92101100	-0.55508900	4.54964200
O	-4.42103800	-3.41076200	-0.75634600
O	-4.45985200	1.26906000	-0.65172500
O	-0.41762400	-1.87762200	2.35708800
O	-2.07564800	-3.00446600	0.55117800
O	-2.69585400	-1.71025400	-1.73024500
O	0.40780400	0.71153200	2.56629400
O	-0.10254600	-4.42694500	1.59700600
O	-2.14167500	-0.62431200	-4.14728900
O	0.43224100	2.75525300	0.82585000
O	-2.12648000	0.90248700	-2.00318600
O	0.45458000	-2.42483400	-0.12992000
O	-1.36964000	4.04002700	2.24723000
O	0.11261200	0.21474500	0.01354500
O	-0.19681400	-0.97449500	-2.31451000
O	2.51520600	1.02066600	0.83642300
O	2.29349800	-0.76777900	-1.19107600
O	-2.14927700	2.26005800	0.35761900
O	-3.95161700	-1.10314500	0.58925100
O	-1.12893300	4.64407900	-0.39724900
O	4.79247400	1.65364400	-0.57373100
H	-1.04704600	0.25558200	5.05351300
Si	-0.77834400	-0.38095600	2.92704300
Si	-3.28378600	-2.31504700	-0.32579300

Si	-0.53215400	-2.92895200	1.09394000
Si	-3.00469000	1.88804800	-1.02258500
H	-4.45950000	0.38468800	-0.21889300
H	-1.99912100	-1.45688500	-4.60889700
H	0.50356400	-4.45868700	2.34364600
Si	-1.79290600	-0.60768500	-2.54721300
Si	0.91866400	1.20669700	1.07055200
H	-1.99986800	4.76674400	2.28860500
Si	0.70325000	-1.00439100	-0.93426100
H	-4.91429400	-3.81841000	-0.03743100
Si	-1.04622700	3.47156000	0.73881400
H	-3.50393700	3.34721200	-2.68189800
H	-1.79381200	4.45985100	-1.08745200
H	-2.18890100	0.94502700	1.69629900
H	-3.38780200	-0.76777200	1.32974300
Mo	3.78864100	0.31657000	-0.45661300
C	4.49701300	-0.11997600	-2.64364800
H	3.57034100	-0.40088100	-3.14650100
H	4.99068500	0.80077200	-2.96254700
C	5.21099200	-1.07458700	-1.93331200
C	4.92051400	-0.91511800	0.47231300
H	4.84680800	-2.10401600	-1.88227400
H	4.40502800	-1.87823400	0.64131500
C	6.26800100	-0.79047500	1.08490900
H	6.76886500	0.14821300	0.81167600
H	6.15123000	-0.80668300	2.18387500
H	6.91525000	-1.64774400	0.82986200
H	6.26985600	-0.92367500	-1.71039400

$TS_{4a\text{-}5}$

O	-2.30813300	0.07185400	2.19715300
O	-3.37976000	3.11462600	-1.92981200
O	-1.03930100	-0.47308600	4.55369200
O	-4.11173000	-3.70851300	-0.76446300
O	-4.52284100	0.95757700	-0.76344300
O	-0.37135300	-1.79065100	2.40406900
O	-1.84602500	-3.09319000	0.60538400
O	-2.48498400	-1.90259700	-1.72390100
O	0.24165500	0.86211200	2.57939200
O	0.33819600	-4.27673800	1.68658100
O	-1.98880700	-0.83918400	-4.16487800
O	0.21568100	2.84503000	0.76883400
O	-2.14426500	0.74244500	-2.06672200
O	0.64141100	-2.32799500	-0.06376400
O	-1.70925900	4.07827700	2.06937600
O	0.07998200	0.26397300	0.03693300
O	-0.05306500	-0.96481600	-2.29363000
O	2.39544700	1.24386900	0.92386500
O	2.36570400	-0.52531000	-1.11226200
O	-2.31617000	2.16025900	0.25209200
O	-3.87295300	-1.34654000	0.53474600
O	-1.41802400	4.56052600	-0.59797700
O	5.04211900	0.60911300	0.90252400
H	-1.20308500	0.33885700	5.04440800
Si	-0.86031800	-0.31696000	2.93334300
Si	-3.07492300	-2.51948200	-0.32494300
Si	-0.31343900	-2.87017400	1.16169700
Si	-3.11533100	1.68096900	-1.12843200
H	-4.46413400	0.08133600	-0.31777600
H	-1.76580800	-1.66876400	-4.59930800

H	0.13192800	-4.52481700	2.59332600
Si	-1.67167400	-0.74747800	-2.56081200
Si	0.78107300	1.33728900	1.08857500
H	-2.38559600	4.76333900	2.05694500
Si	0.79854200	-0.90557400	-0.88545200
H	-4.65985500	-4.07574600	-0.06389000
Si	-1.30316700	3.45624500	0.60305300
H	-3.72326200	3.05057700	-2.82794600
H	-2.07291400	4.31555500	-1.27873600
H	-2.32495100	0.87757500	1.63896100
H	-3.36295100	-0.94056000	1.28007800
Mo	3.79342200	0.51820900	-0.20835900
C	4.39028100	1.75312800	-1.54167300
H	3.76365100	1.95418500	-2.42213200
H	5.30496100	2.35186200	-1.46686500
C	5.16092900	-0.33155300	-2.06665300
C	4.98957700	-1.30077000	-1.06586400
H	4.50330000	-0.34672400	-2.93834500
H	4.15683900	-1.99466200	-1.21312400
C	6.12217300	-1.78539600	-0.20259500
H	6.89319800	-1.01605200	-0.05918400
H	5.77334900	-2.10492400	0.78964600
H	6.59112900	-2.66144700	-0.68352200
H	6.14381900	0.12719100	-2.20946600

$TS_{4a\text{-}4b}$

O	-2.00918600	0.16258300	2.35752900
O	-3.47932500	3.23163500	-1.63626700
O	-0.45969300	-0.43857700	4.51984600
O	-4.36831500	-3.53701800	-0.30537000
O	-4.57256900	1.14059200	-0.30769800
O	-0.15071500	-1.79768400	2.31419600
O	-1.91395700	-3.02650800	0.74532100
O	-2.78900700	-1.81963000	-1.49337300
O	0.61229200	0.81340700	2.37160600
O	0.35006600	-4.31161900	1.49914600
O	-2.57001600	-0.79338100	-3.99484100
O	0.39790700	2.81083500	0.58552200
O	-2.39730200	0.79906700	-1.90575100
O	0.50619100	-2.36722500	-0.25716900
O	-1.27986800	4.10305600	2.14112100
O	0.02914300	0.23691100	-0.11681300
O	-0.41637800	-0.99767000	-2.39480200
O	2.49261400	1.10024600	0.39634400
O	2.15088500	-0.64661400	-1.51852500
O	-2.20117100	2.23284300	0.40687200
O	-3.85041500	-1.18645200	0.92161200
O	-1.33876800	4.61486400	-0.53254000
O	4.45518800	1.57902600	-1.53629500
H	-0.60979000	0.37199100	5.01720400
Si	-0.49658000	-0.30205200	2.88784500
Si	-3.22554300	-2.39760800	-0.02561500
Si	-0.30905600	-2.87379000	1.07507100
Si	-3.18938800	1.78886400	-0.86078000
H	-4.49539000	0.26625700	0.13755900
H	-2.29172700	-1.56335400	-4.50110900
H	0.05287300	-4.68764500	2.33363200
Si	-2.04360900	-0.70815800	-2.44727000
Si	0.91838200	1.27411200	0.81240500
H	-1.91866200	4.81864200	2.22476500

Si	0.61432200	-0.95520300	-1.11140900
H	-4.88378500	-3.81677400	0.45753400
Si	-1.10442500	3.48778600	0.62679200
H	-3.89624200	3.17717200	-2.50358300
H	-2.06750900	4.38854100	-1.14122500
H	-2.06830200	0.95844600	1.78758700
H	-3.23005000	-0.80446900	1.59065800
Mo	3.76367500	0.25607900	-0.80264700
C	4.72809600	-1.24510600	-1.90428200
H	4.22693100	-2.21759300	-1.94180100
H	5.13369200	-0.94113800	-2.87454200
C	5.70704900	-1.07503000	-0.71595900
C	5.03937300	-0.45973100	0.60512200
H	6.02216400	-2.08373900	-0.40808200
H	4.54045100	-1.26740600	1.16801900
C	5.93559300	0.39018300	1.47345600
H	6.39493500	1.21314000	0.90635100
H	5.34953000	0.83357600	2.29239700
H	6.74492700	-0.20994100	1.92542800
H	6.58267900	-0.46968400	-0.98553000

$TS_{7\text{-}8a}$

O	-2.05151500	0.25619000	2.37974500
O	-2.90161700	3.79251100	-1.41668800
O	-0.64676800	-0.75737300	4.48652300
O	-5.12108800	-2.73038600	-0.46297300
O	-4.38393800	1.88332500	-0.20047300
O	-0.59746400	-2.01472400	2.19558200
O	-2.62041600	-2.76595700	0.59819800
O	-3.24616800	-1.28303100	-1.56389900
O	0.64505100	0.39515100	2.40725300
O	-0.82999200	-4.55441000	1.30271400
O	-2.79882200	-0.19018700	-4.00511800
O	0.82410200	2.49521200	0.73943500
O	-2.30958000	1.21519900	-1.83429400
O	-0.11037300	-2.56773000	-0.38118100
O	-0.57131500	4.01765200	2.36058900
O	-0.01511300	0.07552300	-0.10429500
O	-0.74521700	-0.92248800	-2.42656100
O	2.57054900	0.47736900	0.43824000
O	1.84773900	-1.14418200	-1.58551200
O	-1.84212400	2.44614200	0.54939800
O	-4.14330200	-0.59388500	0.89319900
O	-0.51592300	4.64315600	-0.29250400
O	4.45343400	-1.91110600	-1.34939200
H	-0.66108400	0.03554800	5.03212900
Si	-0.65609800	-0.52108700	2.86591400
Si	-3.78104100	-1.85399800	-0.12507600
Si	-1.03061200	-2.97528700	0.92812600
Si	-2.90008700	2.27714300	-0.72844500
H	-4.48117500	0.98767100	0.19850000
H	-2.72826500	-0.98727000	-4.54035100
H	0.06036700	-4.83685200	1.53337000
Si	-2.27795100	-0.30248800	-2.45728100
Si	1.05321400	0.87281400	0.87554700
H	-1.09955600	4.81224600	2.48974700
Si	0.27826200	-1.16637200	-1.16092400
H	-5.58327600	-3.11216900	0.28999800
Si	-0.51923000	3.44449700	0.82022100
H	-3.34919100	3.87880900	-2.26590200

H	-1.28591000	4.60506100	-0.89172400
H	-1.95231200	1.07879900	1.85664900
H	-3.45990000	-0.39489500	1.57926400
Mo	3.57813700	-0.54490400	-0.93717100
C	5.16625300	-0.26843400	0.71917500
H	4.54224000	0.01641700	1.57279100
C	5.53318900	0.79785600	-0.13595400
C	4.36113500	0.76977400	-2.08360200
H	3.95632000	1.79141500	-2.08548600
C	6.05943400	-1.46429300	0.93041300
H	6.63763800	-1.71455800	0.03031500
H	6.76924500	-1.24680800	1.74787000
H	5.48855600	-2.35755500	1.22190700
H	5.13605700	0.56630400	-2.83082700
C	5.25562300	2.23086200	0.24020900
H	4.22749400	2.35478900	0.60782300
H	5.43105200	2.92910100	-0.58981300
H	5.94061800	2.50958300	1.05887700
H	6.42403400	0.62683900	-0.75242700

$TS_{8a\text{-}9}$

O	-2.06642600	0.26046000	2.36700300
O	-3.32202000	3.47400200	-1.56123300
O	-0.55153800	-0.49360500	4.50347000
O	-4.79441200	-3.20674400	-0.24451800
O	-4.56652500	1.48705900	-0.20848400
O	-0.38419700	-1.85460900	2.28444600
O	-2.28047600	-2.90941500	0.74616800
O	-3.10786800	-1.62071400	-1.46600300
O	0.59809300	0.68254600	2.35197700
O	-0.13434400	-4.40254600	1.45863300
O	-2.83539400	-0.60017400	-3.96458800
O	0.56897700	2.68788600	0.56719800
O	-2.47796100	0.95163700	-1.86763300
O	0.16938700	-2.47471700	-0.30026400
O	-0.95755900	4.13688800	2.13986000
O	-0.02529300	0.16708500	-0.14536000
O	-0.68402900	-1.01555900	-2.40431900
O	2.50742300	0.79065300	0.39459000
O	1.93133200	-0.91082500	-1.63106900
O	-2.08260200	2.34870100	0.43916900
O	-4.04981800	-0.91394600	0.97643400
O	-1.01321200	4.62721200	-0.53949500
O	4.46391400	-0.29437500	-2.44594800
H	-0.64872600	0.32081400	5.00752800
Si	-0.59270600	-0.33903100	2.87215000
Si	-3.55301700	-2.16921100	0.01069400
Si	-0.66226600	-2.90848700	1.04746600
Si	-3.14490800	2.00553500	-0.79934600
H	-4.56464000	0.60056300	0.21982000
H	-2.61908800	-1.37892000	-4.48758900
H	-0.43944200	-4.74486700	2.30461700
Si	-2.27734100	-0.57774100	-2.42609500
Si	0.96576500	1.10761100	0.79239200
H	-1.55024600	4.89018700	2.23128700
Si	0.38570900	-1.07924000	-1.15518600
H	-5.30617400	-3.45453100	0.53178600
Si	-0.86550200	3.49290800	0.62901500
H	-3.77791300	3.46497600	-2.41040400
H	-1.78471500	4.47784700	-1.11852600

H	-2.05614200	1.06253500	1.80224600
H	-3.38233200	-0.59016900	1.63121800
Mo	3.59959200	-0.12813800	-1.01670200
C	4.67247800	-1.25573400	0.11474900
H	4.24129600	-1.32439300	1.12780500
C	5.51081800	0.82450000	0.20707200
C	4.82609200	1.71483300	-0.66104500
H	4.17710500	2.47251200	-0.21466100
C	5.79304300	-2.19089300	-0.17195700
H	6.23297500	-2.02423800	-1.16482700
H	6.58083100	-2.13975700	0.59951400
H	5.39440800	-3.22059100	-0.14453900
H	5.28192600	1.96006500	-1.62299100
C	5.44266800	1.00647800	1.70173300
H	4.40851200	1.18751000	2.02663600
H	6.04155700	1.89239500	1.97162500
H	5.85286800	0.14989500	2.25529100
H	6.44057100	0.39683800	-0.18321700

$TS_{8a\text{-}8b}$

O	-2.22664200	0.11894700	2.32928400
O	-3.59421500	3.21279400	-1.67279700
O	-0.74971200	-0.48426500	4.54245100
O	-4.45774300	-3.58648700	-0.42323000
O	-4.70565700	1.10118400	-0.39237900
O	-0.34812500	-1.81851800	2.33851200
O	-2.03628600	-3.05037400	0.68914000
O	-2.87613400	-1.83900400	-1.55984000
O	0.38144000	0.80398100	2.44651500
O	0.20677300	-4.32295700	1.52859300
O	-2.57845300	-0.78536100	-4.04269300
O	0.23049900	2.80260800	0.65929300
O	-2.48782300	0.78949400	-1.93352800
O	0.40832300	-2.36577800	-0.21266400
O	-1.51000100	4.07059800	2.16778900
O	-0.10575700	0.24174600	-0.06182400
O	-0.48232500	-0.98787800	-2.36370600
O	2.34713900	1.09465700	0.57377300
O	2.05034200	-0.58423700	-1.40286200
O	-2.36202400	2.20580000	0.39451300
O	-4.00085900	-1.23610800	0.83249700
O	-1.48264600	4.59566400	-0.50430900
O	4.05063100	1.17784600	-2.21002700
H	-0.89904100	0.32404000	5.04371100
Si	-0.72733400	-0.32841200	2.91177700
Si	-3.33946200	-2.43346200	-0.10531200
Si	-0.45001200	-2.88921000	1.09221700
Si	-3.31354400	1.76709100	-0.90057700
H	-4.63496200	0.22230700	0.04465800
H	-2.30673900	-1.56466100	-4.53818500
H	-0.04994900	-4.66589700	2.39035100
Si	-2.11252600	-0.71011700	-2.47646800
Si	0.77169400	1.27205100	0.90523200
H	-2.16234300	4.77575300	2.23310200
Si	0.48802400	-0.94410800	-1.03960800
H	-4.98113700	-3.88925900	0.32537600
Si	-1.27676000	3.46412000	0.65727300
H	-3.99471300	3.16376400	-2.54808800
H	-2.19615700	4.37311600	-1.13159600
H	-2.27011700	0.92143400	1.76701500

H	-3.40287900	-0.85011000	1.52076400
Mo	3.59526900	0.40504400	-0.80786500
C	4.92041700	-1.09381100	-0.43861200
H	4.55202900	-1.67353700	0.42685000
C	5.83501700	0.10284700	0.10006500
C	4.98747200	1.34965400	0.46241100
H	4.70834300	1.40047400	1.52023000
C	5.52190300	-1.97241700	-1.51299700
H	5.82853000	-1.38989000	-2.39435400
H	6.40790800	-2.51377600	-1.13688100
H	4.79047800	-2.72411900	-1.84517500
H	5.38682500	2.29928800	0.09033800
C	6.62103200	-0.39326100	1.32078300
H	5.94160500	-0.63630000	2.15178600
H	7.31447800	0.38857000	1.66612800
H	7.20162600	-1.29496500	1.07185300
H	6.52278900	0.35770600	-0.72092200

C_2H_4

C	0.00000000	0.00000000	0.66597900
H	0.00000000	0.93101600	1.24174100
H	0.00000000	-0.93101600	1.24174100
C	0.00000000	0.00000000	-0.66597900
H	0.00000000	-0.93101600	-1.24174100
H	0.00000000	0.93101600	-1.24174100

C_3H_6

C	1.29565800	0.14592000	0.00000000
C	0.00000000	0.46918100	0.00000000
H	2.07606700	0.91202400	0.00000000
H	1.62689100	-0.89836800	0.00000000
H	-0.27287000	1.53312400	0.00000000
C	-1.14016100	-0.49848100	0.00000000
H	-0.79196000	-1.54233800	0.00000000
H	-1.78555300	-0.35207900	0.88312700
H	-1.78555300	-0.35207900	-0.88312700

***trans*-2-C_4H_8**

C	-0.54448900	1.88473400	0.00000000
C	-0.54448900	0.38899700	0.00000000
H	-1.07095300	2.28619000	0.88327900
H	0.47753800	2.29322100	0.00000000
H	-1.07095300	2.28619000	-0.88327900
C	0.54448900	-0.38899700	0.00000000
H	-1.53093300	-0.09602500	0.00000000
C	0.54448900	-1.88473400	0.00000000
H	1.53093300	0.09602500	0.00000000
H	1.07095300	-2.28619000	-0.88327900
H	1.07095300	-2.28619000	0.88327900
H	-0.47753800	-2.29322100	0.00000000

References

1 P.-b. Zhou, in *Numerical Analysis of Electromagnetic Fields*, ed. P.-b. Zhou, Springer Berlin Heidelberg, 1993, DOI: 10.1007/978-3-642-50319-1_3, pp. 63-94.

2 L. Petzold, Automatic Selection of Methods for Solving Stiff and Nonstiff Systems of Ordinary Differential Equations, *SIAM J. Sci. Comput.*, 1983, **4**, 136-148.

3 D. E. Mears, Diagnostic criteria for heat transport limitations in fixed bed reactors, *J. Catal.*, 1971, **20**, 127-131.

4 H. S. Fogler, *Elements of chemical reaction engineering*, Prentice Hall, Philadelphia, PA, 5 edn., 2016.

5 F. Gao and L. Han, Implementing the Nelder-Mead simplex algorithm with adaptive parameters, *Comput. Optim. Appl*, 2012, **51**, 259-277.

6 G. Van Rossum and F. L. Drake, *Python 3 Reference Manual*, CreateSpace.

7 P. Virtanen, R. Gommers, T. E. Oliphant, M. Haberland, T. Reddy, D. Cournapeau, E. Burovski, P. Peterson, W. Weckesser, J. Bright, S. J. van der Walt, M. Brett, J. Wilson, K. J. Millman, N. Mayorov, A. R. J. Nelson, E. Jones, R. Kern, E. Larson, C. J. Carey, İ. Polat, Y. Feng, E. W. Moore, J. VanderPlas, D. Laxalde, J. Perktold, R. Cimrman, I. Henriksen, E. A. Quintero, C. R. Harris, A. M. Archibald, A. H. Ribeiro, F. Pedregosa, P. van Mulbregt, A. Vijaykumar, A. P. Bardelli, A. Rothberg, A. Hilboll, A. Kloeckner, A. Scopatz, A. Lee, A. Rokem, C. N. Woods, C. Fulton, C. Masson, C. Häggström, C. Fitzgerald, D. A. Nicholson, D. R. Hagen, D. V. Pasechnik, E. Olivetti, E. Martin, E. Wieser, F. Silva, F. Lenders, F. Wilhelm, G. Young, G. A. Price, G.-L. Ingold, G. E. Allen, G. R. Lee, H. Audren, I. Probst, J. P. Dietrich, J. Silterra, J. T. Webber, J. Slavič, J. Nothman, J. Buchner, J. Kulick, J. L. Schönberger, J. V. de Miranda Cardoso, J. Reimer, J. Harrington, J. L. C. Rodríguez, J. Nunez-Iglesias, J. Kuczynski, K. Tritz, M. Thoma, M. Newville, M. Kümmerer, M. Bolingbroke, M. Tartre, M. Pak, N. J. Smith, N. Nowaczyk, N. Shebanov, O. Pavlyk, P. A. Brodtkorb, P. Lee, R. T. McGibbon, R. Feldbauer, S. Lewis, S. Tygier, S. Sievert, S. Vigna, S. Peterson, S. More, T. Pudlik, T. Oshima, T. J. Pingel, T. P. Robitaille, T. Spura, T. R. Jones, T. Cera, T. Leslie, T. Zito, T. Krauss, U. Upadhyay, Y. O. Halchenko, Y. Vázquez-Baeza and C. SciPy, SciPy 1.0: fundamental algorithms for scientific computing in Python, *Nature Methods*, 2020, **17**, 261-272.

8 C. R. Harris, K. J. Millman, S. J. van der Walt, R. Gommers, P. Virtanen, D. Cournapeau, E. Wieser, J. Taylor, S. Berg, N. J. Smith, R. Kern, M. Picus, S. Hoyer, M. H. van Kerkwijk, M. Brett, A. Haldane, J. F. del Río, M. Wiebe, P. Peterson, P. Gérard-Marchant, K. Sheppard, T. Reddy, W. Weckesser, H. Abbasi, C. Gohlke and T. E. Oliphant, Array programming with NumPy, *Nature*, 2020, **585**, 357-362.

9 M. Newville, T. Stensitzki, D. B. Allen and A. Ingargiola, LMFIT: Non-Linear Least-Square Minimization and Curve-Fitting for Python, 2014, DOI: 10.5281/zenodo.11813.

10 J. Handzlik, K. Kurleto and M. Gierada, Computational Insights into Active Site Formation during Alkene Metathesis over a MoOx/SiO2 Catalyst: The Role of Surface Silanols, *ACS Catal.*, 2021, **11**, 13575-13590.

11 K. Kurleto, F. Tielens and J. Handzlik, Isolated molybdenum (VI) and tungsten (VI) oxide species on partly dehydroxylated silica: A computational perspective, *J. Phys. Chem. C*, 2020, **124**, 3002-3013.

12 A. D. Becke, Density-functional thermochemistry. III. The role of exact exchange., *J. Chem. Phys.*, 1993, **98**, 5648–5652.

13 F. Weigend and R. Ahlrichs, Balanced basis sets of split valence, triple zeta valence and quadruple zeta valence quality for H to Rn: Design and assessment of accuracy, *Phys. Chem. Chem. Phys.*, 2005, **7**, 3297-3305.

14 K. Fukui, The path of chemical reactions-the IRC approach, *Acc. Chem. Res.*, 1981, **14**, 363-368.

15 H. P. Hratchian and H. B. Schlegel, Accurate reaction paths using a Hessian based predictor–corrector integrator, *J. Chem. Phys.*, 2004, **120**, 9918-9924.

16 H. Hratchian and H. Schlegel, Using Hessian updating to increase the efficiency of a Hessian based predictor-corrector reaction path following method, *J. Chem. Theory Comput.*, 2005, **1**, 61-69.

17 Y. Zhao and D. G. Truhlar, The M06 suite of density functionals for main group thermochemistry, thermochemical kinetics, noncovalent interactions, excited states, and transition elements: two new functionals and systematic testing of four M06-class functionals and 12 other functionals, *Theor. Chem. Acc.*, 2008, **120**, 215-241.

18 M. J. Frisch, G. W. Trucks, H. B. Schlegel, G. E. Scuseria, M. A. Robb, J. R. Cheeseman, G. Scalmani, V. Barone, G. A. Petersson, H. Nakatsuji, X. Li, M. Caricato, A. V. Marenich, J. Bloino, B. G. Janesko, R. Gomperts, B. Mennucci, H. P. Hratchian, J. V. Ortiz, A. F. Izmaylov, J. L. Sonnenberg, D. Williams-Young, F. Ding, F. Lipparini, F. Egidi, J. Goings, B. Peng, A. Petrone, T. Henderson, D. Ranasinghe, V. G. Zakrzewski, J. Gao, N. Rega, G. Zheng, W. Liang, M. Hada, M. Ehara, K. Toyota, R. Fukuda, J. Hasegawa, M. Ishida, T. Nakajima, Y. Honda, O. Kitao, H. Nakai, T. Vreven, K. Throssell, J. A. Montgomery, Jr., J. E. Peralta, F. Ogliaro, M. J. Bearpark, J. J. Heyd, E. N. Brothers, K. N. Kudin, V. N. Staroverov, T. A. Keith, R. Kobayashi, J. Normand, K. Raghavachari, A. P. Rendell, J. C. Burant, S. S. Iyengar, J. Tomasi, M. Cossi, J. M. Millam, M. Klene, C. Adamo, R. Cammi, J. W. Ochterski, R. L. Martin, K. Morokuma, O. Farkas, J. B. Foresman and D. J. Fox, Gaussian 16, Revision C.01, Gaussian, Inc., Wallingford CT, 2016.
